# Reactive oxygen species in health and disease

**DOI:** 10.1186/s43556-026-00419-2

**Published:** 2026-03-13

**Authors:** Yaoxing Ren, Jitian Li, Xiaofeng Dai

**Affiliations:** 1 National Local Joint Engineering Research Center for Precision Surgery & Regenerative Medicine, The First Affiliated Hospital of Xi’an Jiaotong University, Xi’an Jiaotong University, Xi’an, 710061 China; 2 School of Future Technology, Xi’an Jiaotong University, Xi’an, 710061 China; 3 Clinical Medical Center of Tissue Engineering and Regeneration, The Third Affiliated Hospital of Henan Medical University, Henan Medical University, Xinxiang, 453000 China

**Keywords:** Reactive oxygen species, Radical, Non-radical, Cancer, Cold atmospheric plasma, Plasma complex medicine

## Abstract

The traditional view of reactive oxygen species (ROS) as uniform toxicants has been superseded by the recognition of a fundamental radical/non-radical dichotomy. As radical and non-radical ROS differ in spatial and kinetic behaviors that dictate cellular impacts, understanding this dichotomy is essential for the design of ROS-targeting therapies. However, the roles of specific ROS types under physiological and pathological conditions remain inadequately defined, hindering precise clinical translation. By organizing ROS sources, neutralizing systems, reaction kinetics, biological effects, and therapeutic strategies along a radical versus non-radical axis, this review clarifies their unique and shared attributes to facilitate effective exploitation for health and disease management. Radical species, being short-lived and membrane-confined, operate locally at near-diffusion-limited rates, whereas non-radical species support compartment-transcending redox communication. Both types mediate beneficial eustress at low physiological levels, suitable for health promotion; yet provoke oxidative distress at high concentrations, forming the basis for numerous therapeutic applications. We examine how this radical versus non-radical dichotomy guides contemporary redox interventions. In health, while low-dose radicals enhance stress resilience and metabolic adaptation, non-radicals regulate physiological plasticity; in disease, radical-focused therapies enable precise cytotoxicity, and non-radical approaches permit spatially programmable signaling. Furthermore, we highlight the promise of hybrid ROS-targeting strategies leveraging their capacity for synchronized and tunable delivery of both radical and non-radical species, enabling broad therapeutic potential. By delineating ROS biology along chemical and spatial principles, this framework advances targeted redox interventions for complex diseases, underscoring the indispensable role of radical processes in oncology.

## Introduction

Reactive oxygen species (ROS) are a class of highly reactive molecules generated through the incomplete reduction of oxygen. They are fundamentally categorized into two groups based on electronic structure, i.e., radical ROS that contain one or more unpaired electrons like superoxide anion (•O_2_^−^) and hydroxyl radical (•OH), and non-radical ROS that lack unpaired electrons such as hydrogen peroxide (H_2_O_2_) and hypochlorous acid (HOCl) [1]. It is critical to note that the definition of a free radical also encompasses reactive nitrogen species (RNS) such as nitric oxide (•NO). The interplay between ROS and RNS can amplify cellular oxidative stress. For example, the rapid reaction between •O_2_^−^ and •NO yields the potent non-radical oxidant peroxynitrite (ONOO^−^), creating a complex landscape of intertwined oxidative and nitrosative signaling.

This radical/non-radical dichotomy of ROS is essential because it maps directly onto their divergent spatial and kinetic behaviors. Prototypically, radical ROS react at near diffusion-limited rates over nanometer-to-submicrometer distances; this confines their immediate, potent effects to structures proximate to their generation, making them ideal for initiating rapid, localized responses [2, 3]. In contrast, non-radical ROS are more stable and can diffuse across membranes via aquaporins, which permits long-range, compartment-to-compartment communication [4, 5], with signaling specificity conferred by structured relay systems like the peroxiredoxin-thioredoxin (PRX-Trx) axis [6–8]. Cells decode these spatial and chemical signals through thiol-based switches like reversible cysteine oxidation in phosphatases and kinases and transcriptional programs like the Keap1-NRF2 pathway, translating redox chemistry into adaptive cellular outcomes [9, 10].

The radical/non-radical duality constitutes a foundational bifurcation in redox biology, with each class fulfilling distinct and non-interchangeable roles. This is exemplified by their divergent functions. While radical ROS are optimized for driving acute, receptor-mediated cellular responses [2, 3], non-radical ROS such as low-flux H_2_O_2_ sustain the nuanced signaling required for metabolic reprogramming and plasticity, a functional dichotomy central to both health and disease [4, 5]. This specialization is also evident at the systems level. For instance, immune cells deploy a potent radical-to-non-radical oxidant cascade (•O_2_^−^ → H_2_O_2_ → HOCl/ONOO^−^) for host defense, while generic cellular homeostasis relies on non-radical H_2_O_2_ to fine-tune kinase/phosphatase networks governing growth and adaptation [11–13]. Therefore, recognizing this chemical and functional distinction is not merely academic but imperative. It is the intrinsic properties of each ROS class that dictate their specific biological roles, and this understanding is the essential prerequisite for the rational design of targeted strategies to harness or inhibit ROS for precise interventions in human health and disease management.

By systematically organizing the biology of ROS, from their generation and clearance to their chemical behavior, physiological roles in signaling and homeostasis, and their pathogenic contributions across diseases, this review establishes a conceptual framework that maps these multifaceted functions onto the fundamental radical versus non-radical dichotomy. This radical/non-radical axis critically informs the design of species-matched therapeutic interventions, where the distinct spatial scale and reaction mode of each ROS class dictate their optimal therapeutic application. Building on this framework, the review further proposes and highlights innovative hybrid-source ROS technologies as a frontier in redox medicine, opening new avenues for diversified and precisely calibrated applications across the spectrum of human health maintenance and disease management.

## Life cycle and chemical nature of ROS

### Generation and reactivity of radical ROS

**Fig. 1:**
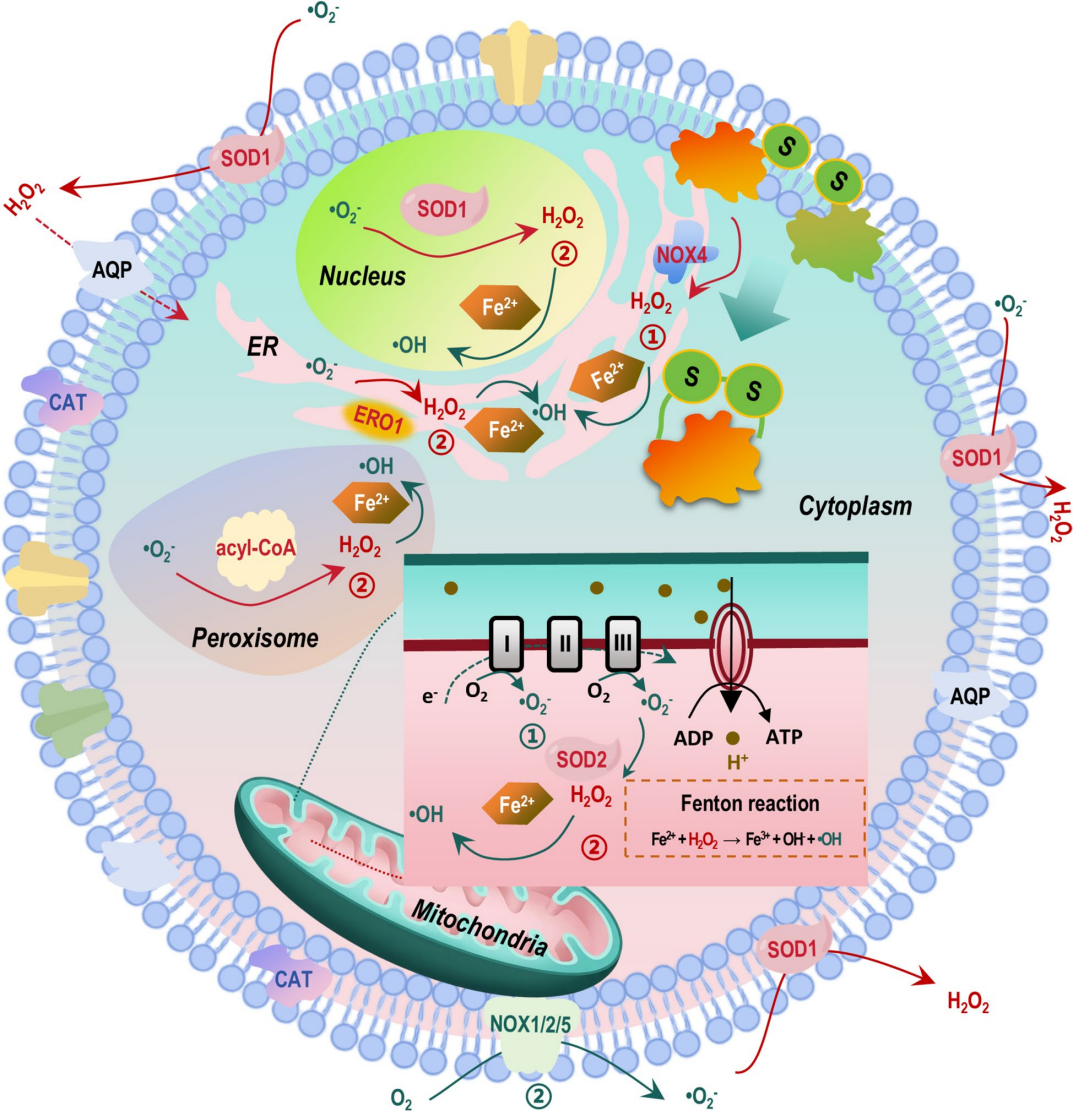
Primary endogenous and exogenous generation sources of radical versus non-radical ROS. Radicals and non-radicals are generated via one-electron and two- or multi-electron processes, respectively, which are interconnected and mutually convertible. Generation of the radical •O_2_^−^ occurs primarily via two distinct mechanisms. The first and major source is the reduction of O_2_ to •O_2_^−^ within the electron transport chain (mitochondria) due to electron leakage, a process accelerated under conditions of a high membrane potential or during reverse electron transfer (① in blue); the second source involves NOX1/2/5 (cell membrane) that directly convert O_2_ to form •O_2_^−^ (② in blue). Production of the non-radical H_2_O_2_ relies on two processes, i.e., direct generation of •O_2_^−^ as a byproduct of protein disulfide bond formation by NOX4 (endoplasmic reticulum) (① in red), and the conversion of •O_2_^−^ to H_2_O_2_ as catalyzed via SOD1 (cytosol and nucleus) and SOD2 (mitochondria) or during metabolic reactions by oxidases like acyl-CoA oxidase (peroxisome) (② in red). Blue and red represent radical and non-radical ROS, respectively. Generation of the radical •OH occurs through the Fenton reaction, where H_2_O_2_ is converted to •OH in the presence of Fe^2+^. Additional abbreviations: ER, endoplasmic reticulum; ETC, electron transport chain; Fe^2+^, ferrous iron; H_2_O_2_, hydrogen peroxide; NOX, NADPH oxidase; O_2_, molecular oxygen; PM, plasma membrane; RET, reverse electron transfer; ROS, reactive oxygen species; SOD, superoxide dismutase; •O_2_^−^, superoxide anion; •OH, hydroxyl radical

Radical ROS operate through a unified chemical logic centered on one-electron transfer (Fig. 1). That is, electrons are sequentially transferred from donors to O_2_, forming **•**O_2_^−^, or H_2_O_2_ is cleaved via Fenton or pseudo-Fenton reactions in the presence of transition metals to generate •OH. This mechanism confers an inherent tendency to initiate chain reactions and achieve localized amplification within the microenvironment [14, 15]. The one-electron transfer principle allows rapid radical generation and propagation in nanodomains containing redox-active metals and peroxides, resulting in high reactivity but spatially confined action in vivo [14, 16].

Complex I (site I_Q_) and Complex III (site Q_o_) are subjected to single-electron leakage under conditions of high membrane potential or reverse electron transport (RET), directly reducing O_2_ to •O_2_^−^ in mitochondria (Fig. 1). The topology of this release, whether into the matrix or intermembrane space, determines subsequent reaction pathways and target accessibility, establishing these sites among the best-characterized endogenous radical sources in mammalian cells [16–21]. Matrix-released •O_2_^−^ is rapidly converted to H_2_O_2_ by superoxide dismutase 2 (SOD2), after which H_2_O_2_ diffuses into the intermembrane space allowing its engagement with distinct targets and scavenging systems (Fig. 1). Electron backflow during RET further amplifies I_Q_ output, leading to site-specific physiological or pathological outcomes [17, 21]. Although SOD2 rapidly converts •O_2_^−^ into H_2_O_2_, redirecting the flux into a non-radical pathway, such a downstream conversion does not negate the radical nature of the initial species or its local near-field reactivity [6, 7]. Thus, the radical ROS acts as the primary local threshold trigger, while the resulting H_2_O_2_ propagates the signal outward through the enzyme-regulated relay systems, extending its reach and duration [16, 18].

As a second major one-electron pathway occurring via the plasma and endomembrane systems, NADPH oxidase isoforms 1/2/5 (NOX1/2/5) reduce O_2_ to •O_2_^−^ near membranes and form ‘on-site generation, on-site action’ radical microdomains (Fig. 1). These domains convert extracellular ligand binding and immune receptor activation into localized radical pulses [22, 23]. As NOX complexes assemble within cell receptor or lipid rafts, the resultant radicals act on nearby targets before dismutation or clearance, enabling precise, spatially confined signaling with limited duration [22].

These radical pulses can serve as upstream triggers that initiate downstream H_2_O_2_ signaling and clearance networks, following a classic ‘radical initiation-chain propagation’ cycle that amplifies the impact of local oxidative events to cells [14, 16]. Alternatively, under conditions of metal enrichment or peroxide accumulation, •O_2_^−^ may be converted locally to •OH, driving reactions toward irreversible damages; and these chain reactions persist at membrane interfaces until terminated by antioxidants or substrate depletion, demonstrating the efficiency of radical-mediated lipid damage within the confined region (Fig. 1) [14, 16]. Thus, the local availability of metals and peroxides determines whether radicals initiate reversible H_2_O_2_-mediated signaling or escalate into •OH-driven oxidative injury [14, 24]. This receptor- and synapse-localized one-electron radical circuit accounts for the rapid, spatially confined chemistry characteristic of early inflammatory and innate immune responses, as well as their tight integration with redox-sensitive signaling nodes [9, 22, 23]. Within these microdomains, redox-sensitive phosphatases and adaptor proteins serve as immediate sensors of radical activity, directly coupling chemical events with signaling outcomes without reliance on long-range diffusion [9].

Exogenous inputs also operate via one-electron mechanisms that integrate with and amplify endogenous redox networks. That is, ionizing radiation generates •OH and hydrated electrons (e_aq_^−^) through water radiolysis within femtoseconds to nanoseconds, followed by secondary species over microseconds to milliseconds. This layered chemistry follows a ‘radicals first, derivatives later’ sequence [25, 26]. While early •OH inflicts clustered DNA and lipid damage, subsequent species including H_2_O_2_ propagate responses into metabolic and inflammatory pathways [25]. For instance, paraquat undergoes redox cycling at mitochondrial site I_Q_, where it repeatedly transfers single electrons to sustain •O_2_^−^ production and amplifies pre-existing mitochondrial generation; such a persistent recycling continuously replenishes the radical pool, overwhelming local dismutation capacity that explains the marked toxicity of paraquat to mitochondria [27]. Particulate matter (PM) and cigarette smoking are known to enhance radical generation and chain reactions at epithelial-immune interfaces through pre-formed radicals and NOX induction. This creates a synergistic pattern in which exogenous one-electron inputs amplify endogenous radical production [28, 29]. Pre-existing radicals and metals in PM, combined with stimulus-driven NOX activation, collectively increase the frequency of initiation and extent of propagation for lipid and protein oxidation in barrier tissues [28, 29]. In metal-enriched microenvironments, Fenton and pseudo-Fenton reactions redirect H_2_O_2_ back into the radical chemistry, generating local •OH. Damage patterns are determined by the spatial co-localization of H_2_O_2_ with redox-active iron or copper pools, resulting in highly confined, poorly buffered oxidative events once initiated [14]. This mechanism confers extreme site specificity to oxidative injury, as observed in ischemia–reperfusion and focal toxicology, and serves as the terminal radical executor in the one-electron pathway [14].

### Generation and reactivity of non-radical ROS

Non-radical ROS are formed via two- or multi-electron processes or energy transfer at their sources, resulting in species whose biological roles are defined by selectivity, reversibility, and diffusibility, rather than the rapid, proximity-driven chain propagation typical of radicals [5, 7, 30] (Fig. 1). Functionally, these oxidants convey information through reversible cysteine modifications and controlled diffusion, moderated by enzymatic and membrane transport systems. Consequently, signal intensity and duration are governed by the capacity of peroxidase and channel networks, not by radical chain kinetics [5, 7, 30].

In the mitochondria, a measurable steady-state efflux of H_2_O_2_ occurs even under resting conditions, which is derived from •O_2_^−^ dismutation by SOD2 and intercompartmental exchange (Fig. 1). This non-radical-enabled phenotype engages PRX-mediated thiol oxidation and provides a sustained, tunable H_2_O_2_ background that supports longer-range signaling and metabolic adaptation [7, 15–18, 20, 21]. Matrix PRX systems serve as both sinks and relays, shaping the amplitude and frequency of oxidation waves. Live-cell imaging with sensors like HyPer7 could capture second-to-minute H_2_O_2_ dynamics that was correlated with cellular protrusion behavior [31], underscoring the role of reaction–diffusion mechanisms in spatiotemporal signal control [25–27, 31–33].

In the endoplasmic reticulum (ER), oxidative protein folding through the ER oxidoreductin 1 (ERO1)- protein disulfide isomerase (PDI) axis continuously generates H_2_O_2_, which is locally contained and recycled by peroxiredoxin 4 (PRDX4) and the glutathione (GSH)/thioredoxin (Trx) system (Fig. 1). This confinement limits oxidative events to the ER lumen, maintaining protein-folding fidelity and regulating the timing of crosstalk with cytosolic redox pathways [34, 35]. Trans-ER communication is further facilitated by aquaporins (AQPs) such as AQP11, and disruption of the folding machinery such as *PDI* depletion reduces ER-derived H_2_O_2_ leakage to the nucleus and mitigates the chance of generating cell senescence [36]. These findings underscore how the spatial organization of H_2_O_2_ production, scavenging, and transport mechanisms collectively govern redox signaling [36–38].

In peroxisomes, oxidases such as acyl-CoA oxidase directly generate H_2_O_2_, while catalase (CAT) and the PRX-Trx system within the same compartment clear and relay oxidizing equivalents (Fig. 1). This co-localization of production and clearance machinery forms a self-buffered microenvironment that serves as a major source of diffusible H_2_O_2_ [39]. The resulting H_2_O_2_ diffusion profile is both stable and tunable, which is in consistent scale with the β-oxidation flux [39, 40]. That is, while CAT limits concentration peaks, the PRX-Trx axis converts H_2_O_2_ into directed redox relays, preventing its leakage and possible undesirable oxidative stress [39].

In addition, unlike NOX1/2/5 that primarily release •O_2_^−^ requiring additional dismutation to form H_2_O_2,_ NOX4, predominantly localized in the ER [41], directly generates H_2_O_2_ (Fig. 1). This has positioned NOX4 as a source of diffusible, non-radical signaling molecule rather than a radical initiator [22, 23]. Due to the relatively constitutive activity and the membrane permeability of H_2_O_2_, NOX4-derived signals can integrate across subcellular compartments to modulate transcriptional responses for adapted metabolic homeostasis [32, 33].

Exogenous inputs such as photochemical and late radiolytic processes also contribute to non-radical oxidants. Specifically, type-II energy transfer (a photophysical mechanism in which an excited photosensitizer molecule directly transfers energy to molecular oxygen) via endogenous or exogenous photosensitizers generates singlet oxygen (^1^O_2_), and UVA can excite endogenous chromophores to produce ^1^O_2_. Although being a non-radical, ^1^O_2_ is highly reactive and responsible for the efficacy and spatial precision of PDT [42, 43]. Since its formation is confined to photosensitizer locations within membranes or organelles, ^1^O_2_ acts as a site-locked effector with a short lifetime that imposes strict spatial constraints. This has enabled its selective target ablation with minimal adverse effects when light delivery and sensitizer localization are well controlled. Furthermore, while early •OH inflicts clustered molecular damage, subsequent H_2_O_2_ propagates signaling over minutes to hours, activating redox-sensitive transcriptional and paracrine programs that influence tissue phenotypes [25, 26]. Thus, non-radical species such as H_2_O_2_ accumulate and contribute to downstream redox responses in the microsecond-to-millisecond window following the initial radical burst from ionizing radiation, reflecting a temporal hierarchy of ‘radicals first, non-radicals later [25, 26].

In summary, non-radical pathways operate in parallel to, not merely downstream of, radical chemistry. While H_2_O_2_ exemplifies selective, reversible, and diffusible signaling, ^1^O_2_ represents a short-range, highly reactive effector. Together, they functionally complement the radical paradigm of rapid, localized chain amplification [5, 7, 22, 30, 35, 39, 42, 43]. From this perspective, mitochondrial H_2_O_2_ efflux, ER oxidative folding, peroxisomes, and NOX4 serve as context-dependent sources for H_2_O_2_ signaling, whereas peroxiporins and luminal scavengers govern the spatial range and fidelity of signal propagation [32–35, 39, 44, 45].

### Clearance of excess ROS

**Fig. 2:**
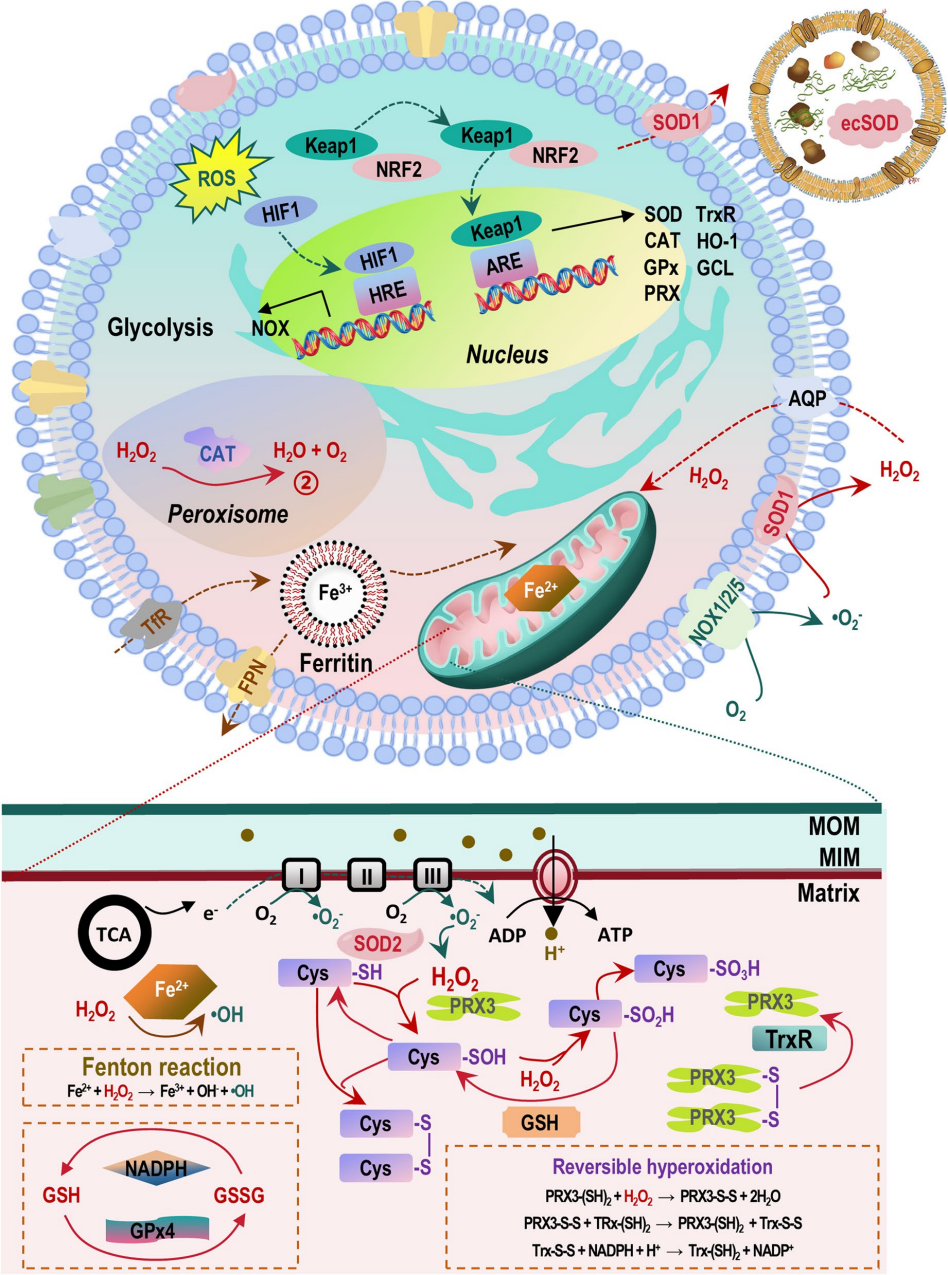
A hierarchical and integrated model of the cellular antioxidant defense network. This schematic illustrates the multi-layered ‘downgrade-then-clear’ principle governing redox homeostasis. The system initiates with SODs, the primary gatekeepers that catalyze the dismutation of •O_2_^−^ into H_2_O_2_ and molecular oxygen. Strategic compartmentalization ensures this conversion occurs at the source of radical generation: SOD1 is localized predominantly in the cytosol and mitochondrial intermembrane space, while SOD2 is exclusively within the mitochondrial matrix. This compartment-specific activity spatially defines the origin of downstream H_2_O_2_ signaling. The produced H_2_O_2_ is subsequently managed by a tiered enzymatic clearance system. The PRX family forms the kinetic frontline, rapidly scavenging the majority of newly formed H_2_O_2_ with high affinity. Their activity is dynamically regulated by a reversible hyperoxidation cycle; under physiological flux, PRXs are active, but a surge in H_2_O_2_ leads to transient inactivation of their catalytic cysteine, acting as a molecular floodgate to permit H_2_O_2_-mediated signal transduction. The enzyme sulfiredoxin repairs these hyperoxidized PRXs, restoring activity. Most PRXs are functionally dependent on the dedicated Trx reduction system, comprising Trx, TrxR, and NADPH, which continuously regenerates active PRXs, positioning the Trx system as a central redox hub. For bulk clearance, high-capacity enzymatic systems are engaged. CAT, primarily located in peroxisomes, directly decomposes H_2_O_2_ into water and oxygen. Parallel to this, the GPx family, utilizing reduced GSH as an electron donor, reduces H_2_O_2_ and organic hydroperoxides. Among these, GPx4 is uniquely critical due to its specificity for reducing complex lipid hydroperoxides. The GSH system, maintained by GSH reductase using NADPH to recycle oxidized glutathione (GSSG) back to GSH, serves as the major non-enzymatic redox buffer. A fundamental preventive defense layer is the meticulous regulation of transition metals, specifically the iron (Fe) cycle. Proteins like ferritin sequester iron in a non-reactive, stored form, minimizing the labile iron pool. This is vital because free Fe^2+^ catalyzes the Fenton reaction, converting H_2_O_2_ into the highly destructive •OH. Iron homeostasis, involving TfR, ferritin, and FPN, thus works in concert with enzymatic clearance to prevent the initiation of radical chain reactions. The entire defense network is subject to long-term adaptive control by master transcriptional programs. The Keap1-NRF2 pathway is the preeminent coordinator: under oxidative stress, NRF2 is stabilized, translocates to the nucleus, and binds the ARE, inducing a vast cytoprotective gene program including SOD, CAT, GPx, PRX, TrxR, and enzymes for GSH synthesis like GCL. Simultaneously, the HIF pathway, activated under low oxygen tension, exerts profound influence by driving metabolic reprogramming and transcribing a context-dependent mix of pro- and antioxidant genes, such as NADPH oxidase (NOX) subunits, thereby shaping the cellular redox landscape. This integrated model depicts how rapid enzymatic reactions, metal homeostasis, and adaptive gene regulation coalesce to neutralize ROS, preserve redox signaling, and maintain cellular integrity. Additional abbreviations: ARE, antioxidant response element; CAT, catalase; Fe^2+^, ferrous iron; FPN, ferroportin; GCL, glutamate–cysteine ligase; GPx, glutathione peroxidase; GR, glutathione reductase; GSH, reduced glutathione; GSSG, oxidized glutathione; H_2_O_2_, hydrogen peroxide; HIF, hypoxia-inducible factor; IMS, intermembrane space; Keap1, Kelch-like ECH-associated protein 1; MIM, mitochondrial inner membrane; MOM, mitochondrial outer membrane; NADPH, reduced nicotinamide adenine dinucleotide phosphate; NOX, NADPH oxidase; NRF2, nuclear factor erythroid 2–related factor 2; PRX, peroxiredoxin; ROS, reactive oxygen species; SOD, superoxide dismutase; SRX, sulfiredoxin; TfR, transferrin receptor; Trx, thioredoxin; TrxR, thioredoxin reductase; •O_2_^−^, superoxide anion; •OH, hydroxyl radical

The cellular defense against oxidative damage is orchestrated by a sophisticated, multi-layered clearance system designed to maintain redox homeostasis. This system operates on a fundamental ‘downgrade-then-clear’ principle to neutralize the threat posed by ROS [6] (Fig. 2). The first critical line of defense involves the rapid conversion of highly reactive, short-lived radical species into more stable, manageable intermediates. This task is performed by SODs, which catalyze the dismutation of the •O_2_^−^ into H_2_O_2_ and molecular oxygen. By executing this conversion, SODs serve as the initial gatekeepers, mitigating near-field radical damage at its very source and transforming a potentially destructive radical pulse into a diffusible non-radical signal that can be processed with greater control [6]. The strategic subcellular localization of SOD isoforms, with SOD1 predominantly in the cytosol and the intermembrane space, and SOD2 exclusively in the mitochondrial matrix, ensures this conversion occurs precisely where superoxide is generated, such as at mitochondrial electron transport chains. This compartmentalization dictates the spatial origin of subsequent H_2_O_2_ signaling, and alterations in the expression of these isoforms can actively rewire redox communication between cellular compartments, influencing metabolic and survival pathways [46].

The H_2_O_2_ produced by SODs is then processed by a tiered enzymatic system that balances the need for rapid detoxification with the preservation of H_2_O_2_'s role as a signaling molecule. The PRX family constitutes the kinetic frontline of this defense. PRXs eliminate the vast majority (> 80–90%) of newly formed H_2_O_2_ with extraordinary speed, acting as high-affinity peroxidases (Fig. 2) [47]. Their function is dynamically regulated through a reversible hyperoxidation mechanism. At physiological or moderately elevated H_2_O_2_ fluxes, PRXs are highly active. However, when H_2_O_2_ levels surge, their catalytic cysteine residue becomes hyperoxidized, leading to a transient inactivation. This acts as a built-in molecular ‘brake’ or floodgate, preventing the PRX system from scavenging all H_2_O_2_ and thus allowing necessary signal transduction to downstream targets, such as specific transcription factors [48]. The enzyme sulfiredoxin subsequently repairs these hyperoxidized PRXs, restoring their activity. This cyclical oxidation-and-repair mechanism creates a tunable temporal rheostat, generating precise windows of opportunity for H_2_O_2_-mediated signaling before clearance resumes [49]. The importance of PRXs is compartment-specific. For instance, PRX3 is responsible for the majority of H_2_O_2_ clearance within the mitochondrial matrix, an organelle highly vulnerable to oxidative and nitrative stress. PRX3’s ability to also efficiently reduce ONOO^−^ at a remarkable rate constant (~ 10^7^ M^−1^·s^−1^) is crucial for protecting mitochondrial integrity and function [47, 50]. The activity of most PRXs is entirely dependent on a dedicated reductant system, the Trx system. This system, comprising Trx, thioredoxin reductase (TrxR), and NADPH, continuously reduces the oxidized (disulfide) form of PRXs back to their active state, completing their catalytic cycle. The Trx system itself is a central hub of redox regulation, with its own redox status influencing numerous cellular processes beyond antioxidant defense.

For bulk clearance, especially when H_2_O_2_ production exceeds the PRX system’s capacity or during sustained oxidative stress, high-capacity enzymatic sinks take over. CAT, primarily located in peroxisomes, catalyzes the direct decomposition of H_2_O_2_ into water and oxygen, serving as a high-throughput overflow mechanism [47]. Parallel to this, the glutathione peroxidase (GPx) family provides another major route for H_2_O_2_ and organic hydroperoxide reduction (Fig. 2). GPx enzymes utilize reduced GSH as an essential electron donor, converting it to oxidized glutathione (GSSG) in the process. Among GPx isoforms, GPx4 holds a unique and critical position due to its membrane association and specificity for reducing complex lipid hydroperoxides within cell membranes. This function makes GPx4 the primary enzymatic barrier against ferroptosis, an iron-dependent form of regulated cell death driven by uncontrolled lipid peroxidation. The loss of GPx4 function rapidly unleashes lethal lipid peroxidation cascades, even under moderate oxidative conditions [51]. The GSH/GSSG couple is, therefore, far more than a simple cofactor; it represents the cell’s most abundant non-enzymatic redox buffer. The ratio of GSH to GSSG is a fundamental indicator of the cellular redox environment. Maintaining a high GSH pool is essential for GPx activity, for direct radical scavenging, and for the function of GSH S-transferases in detoxification. This is achieved by GSH reductase, which uses NADPH to regenerate GSH from GSSG, linking antioxidant capacity directly to cellular metabolic status.

Beyond these core enzymatic and non-enzymatic systems, ROS regulation extends to extracellular spaces and is subject to long-term adaptive control. Dedicated enzymes like NADPH oxidases (NOXs) are not clearance systems per se but are crucial regulators of ROS levels. NOXs are transmembrane proteins that deliberately generate •O_2_^−^ (and subsequently H_2_O_2_) in specific extracellular or organellar compartments (Fig. 2) [52–54]. This controlled production is vital for physiological processes such as microbial killing in phagocytes and cellular signaling in various tissues. The interplay between NOX-derived ROS and the antioxidant systems described above determines localized redox states. Furthermore, cells communicate their redox status and influence their microenvironment through the secretion of antioxidants (e.g., extracellular superoxide dismutase, ecSOD) and the release of redox-active cargo, including proteins, lipids, and miRNAs, packaged within exosomes (EXOs) [55, 56]. These EXOs can deliver antioxidant or pro-oxidant messages to neighboring or distant cells, playing a role in both tissue homeostasis and disease progression.

To adapt to chronic or escalating oxidative stress, cells activate master transcriptional programs that globally upregulate their defensive capabilities. The Keap1-NRF2-ARE pathway is the preeminent coordinator of this adaptive response (Fig. 2). Under basal conditions, NRF2 is bound by Keap1 and targeted for degradation [57, 58]. Oxidative or electrophilic stress modifies Keap1, leading to NRF2 stabilization, nuclear translocation, and binding to the Antioxidant Response Element (ARE) [59, 60]. This induces the transcription of a vast network of cytoprotective genes, including those encoding SOD, CAT, PRX, TrxR, GPx, heme oxygenase-1 (HO-1), and enzymes required for GSH synthesis (e.g., glutamate-cysteine ligase) [61–63]. HO-1 is particularly noteworthy for its dual role in degrading pro-oxidant heme and producing antioxidant biliverdin/bilirubin and carbon monoxide. The Hypoxia-Inducible Factor (HIF) pathway, activated under low oxygen tension (hypoxia), also exerts profound influence on redox balance. HIF-1α stabilization leads to metabolic reprogramming (e.g., a shift towards glycolysis) that can indirectly affect mitochondrial ROS production [64]. Moreover, HIF target genes include both pro-oxidant factors (like NOX subunits) and antioxidant enzymes, illustrating the complex, context-dependent role of HIF in shaping the cellular redox landscape [65, 66]. Finally, a critical layer of defense exists in the meticulous regulation of transition metals, particularly iron. Proteins like ferritin sequester iron in a non-reactive, stored form through its ferroxidase activity, maintaining a minimal ‘labile iron pool’ [67]. This is vital because free ferrous iron (Fe^2+^) catalyzes the Fenton reaction, where H_2_O_2_ is converted into the extremely destructive •OH. Thus, robust iron homeostasis, managed by networks involving transport (transferrin receptor, TfR), storage (ferritin), and export (ferroportin, FPN), works in concert with enzymatic antioxidants to prevent the initiation of the most damaging radical chain reactions, the failure of which is a direct pathway to ferroptosis [68, 69].

In summary, the ROS clearance system is an exquisitely coordinated network spanning immediate enzymatic conversion, dynamic signal modulation, high-capacity scavenging, intercellular communication, and genomic adaptation, all working in concert to preserve cellular integrity and function.

## Physiological roles of ROS in cellular signaling and homeostasis

**Fig. 3:**
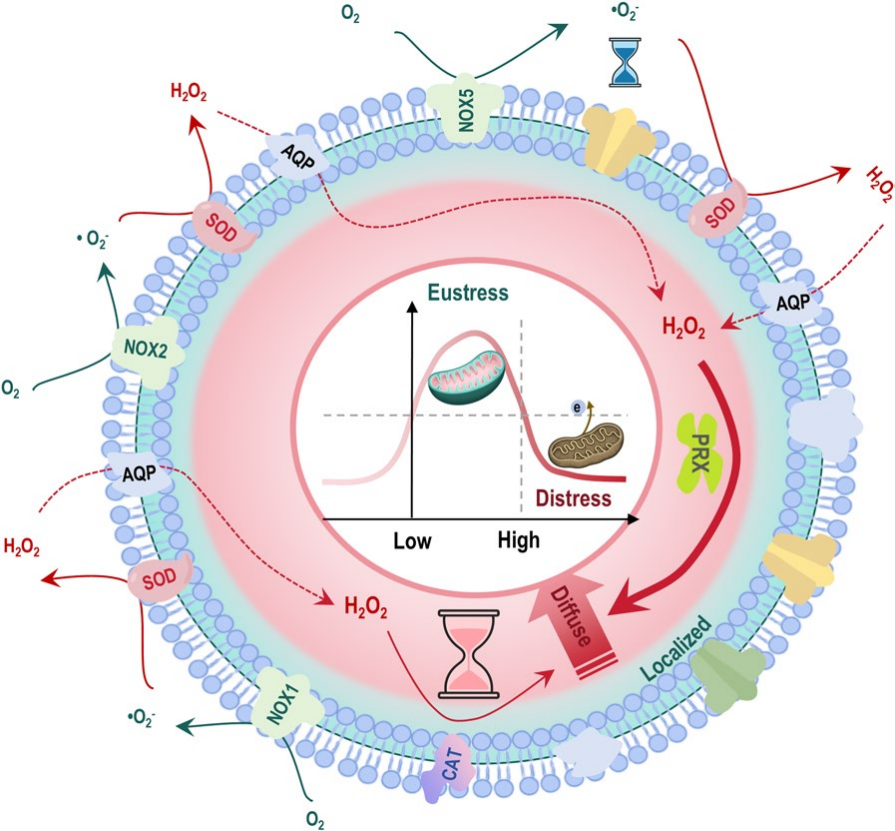
Chemical reaction properties of radical versus non-radical ROS. Radical and non-radical ROS operate over distinct spatiotemporal scales to mediate redox signaling. Radicals like •O_2_^−^ and •OH are short-lived, acting as nanoscale, proximity-limited triggers through rapid electron transfer at membrane receptors or metal centers. In contrast, non-radicals like H_2_O_2_ serve as micrometer-scale messengers, propagating via enzymatic relay systems and controlled diffusion for targeted, sustained signaling. Both classes exhibit a dose-dependent duality. That is, they lead to oxidative eustress at low levels as a result of mitohormesis, but induce oxidative distress at high concentrations due to widespread damage created. Thus, while radicals initiate localized bursts, non-radicals integrate these signals and enable a sophisticated redox language for physiological regulation and therapeutic targeting. Blue and red represent radical and non-radical ROS, respectively. Additional abbreviations: H_2_O_2_, hydrogen peroxide; ROS, reactive oxygen species; •O_2_^−^, superoxide anion; •OH, hydroxyl radical

ROS orchestrate cellular signaling and homeostasis through a sophisticated division of labor defined by their distinct chemical properties and spatiotemporal dynamics. This system hinges on a fundamental binary between the fast, localized activity of radical ROS and the stable, diffusible nature of non-radical ROS. Radical species, such as •O_2_^−^ and •OH, possess unpaired electrons, granting them extremely short lifespans (nanoseconds to microseconds) and confining their reactivity to nanometer-scale ‘reaction–diffusion microdomains’ near their site of generation [5, 8, 30] (Fig. 3). This spatial constraint necessitates that they act as proximity-limited triggers, initiating signals through rapid electron transfer reactions at local targets like membrane receptors or metal centers, rather than as long-range messengers.

In stark contrast, non-radical ROS, e.g., H_2_O_2_, are chemically stable and membrane-permeable, enabling them to function as long-range, diffusible redox messengers. Their physiological signaling is not governed by random diffusion but is meticulously sculpted by enzymatic systems. A core mechanism is the PRX-Trx relay, which acts as a high-fidelity signal transduction circuit [70]. At low fluxes, PRXs rapidly absorb H_2_O_2_ and pass oxidizing equivalents via Trx to specific client proteins (e.g., phosphatases, transcription factors), creating controlled, micrometer-scale gradients that translate a global oxidant presence into precise pathway outputs. This process is further regulated by peroxiporins (e.g., AQP3), which gate transmembrane H_2_O_2_ flux to shape inter-organelle communication [8].

Despite their divergent mechanisms, both radical and non-radical ROS share a critical dose-dependent duality. At low, physiologically buffered concentrations, they mediate oxidative eustress, a form of beneficial stress that drives adaptive responses in cell survival, metabolic plasticity, and immune homeostasis. This adaptive signaling is enabled by reversible post-translational modifications, such as cysteine oxidation, and structured relay systems that prevent collateral damage. However, when production overwhelms the capacity of these containment and buffering systems (e.g., PRX saturation), the same species induce oxidative distress, leading to irreversible macromolecular damage, metabolic dysfunction, and pathological inflammation. Therefore, cellular redox homeostasis is not a simple state of low oxidation but a dynamic balance maintained by the compartmentalized, enzyme-guided activities of both radical triggers and non-radical relays, whose biological outcomes are exquisitely dependent on flux magnitude and duration.

### Physiological roles of radical ROS in cellular signaling and homeostasis

A core principle governing the physiological roles of radical ROS, e.g., •O_2_^−^, is their spatially and temporally constrained nature. Under physiological pH, with the acid dissociation constant (pKa) being approximately 4.8, •O_2_^−^ exists predominantly as an anion with limited membrane permeability, confining it largely to its site of generation. Its high reactivity and nanosecond-scale scavenging further restrict its activity to nanometer-submicrometer microdomains, preventing long-range diffusion [15, 71] (Fig. 3). This spatial restriction establishes •O_2_^−^ as a precise near-field trigger. The biological function of this localized radical flux is critically shaped by SOD1 and SOD2, which catalyze the dismutation of •O_2_^−^ to H_2_O_2_ within microseconds. This reaction acts as a fundamental redox relay system, converting a short-lived, site-confined radical signal into a longer-lived, diffusible H_2_O_2_ wave [16, 17, 72] (Fig. 3). The resulting H_2_O_2_ then serves as a secondary messenger, decoded by downstream effectors like thiol peroxidases to regulate adaptive cellular responses. This model is exemplified by mitochondrial site I_Q_, where discrete •O_2_^−^ production during forward or RET is compartmentalized; and site-specific inhibitors (S1QELs) can suppress this •O_2_^−^/H_2_O_2_ output without affecting basal respiration, demonstrating that the site of generation dictates specific signaling outcomes [24, 72]. Thus, physiological radical ROS flux operates within a tightly controlled spatial relay system (•O_2_^−^ → H_2_O_2_), enabling localized redox cues to be transduced into adaptive signals for cell survival, metabolic plasticity and immune homeostasis, while loss of this control drives pathological, switch-like transitions toward cell death, metabolic dysfunction and pathological inflammation.

#### Radical ROS in cell fate: survival vs. death

**Fig. 4:**
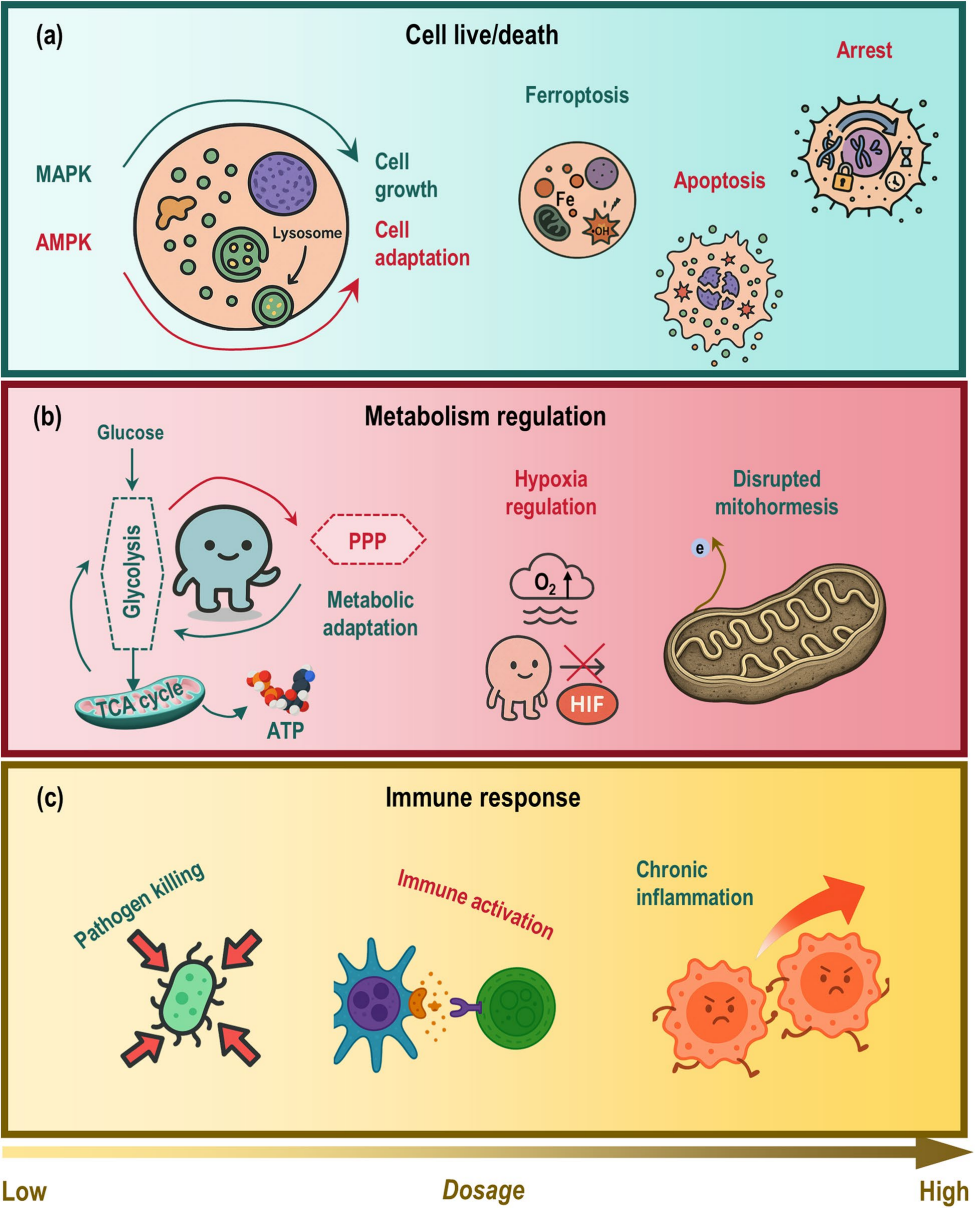
Physiological roles of radical versus non-radical ROS in cellular signaling and homeostasis. Radical and non-radical ROS function as biphasic regulators of cell fate, primarily cell live/death switch, immune response and metabolism regulation, through distinct spatiotemporal mechanisms. Both radicals and non-radicals exhibit dose-dependent duality, i.e., while they encode both survival and termination cues within a unified programmed framework, support physiological immune defense and metabolic plasticity under low concentrations, they provoke cell death, chronic inflammation, and disrupted mitohormesis when in excess. **a** For cell live/death, at physiological levels, radical ROS like •O_2_^−^ promote mitogenic signaling via reversible oxidation of phosphatases like PTPs, activating MAPK pathways and supporting proliferation, while non-radical H_2_O_2_ acts as a diffusible messenger to fine-tune kinases such as AMPK for sustained adaptive responses. At pathological levels, exceeding critical redox thresholds cells can tolerate triggers cytotoxicity. While radical species drive ferroptosis, excessive H_2_O_2_ arrests cell growth and induces apoptosis. **b** For immune response, at physiological levels, nanoscale-confined radical species like •O_2_^−^ serve as precise triggers, directly mediating pathogen killing in phagosomes. In parallel, non-radical ROS like H_2_O_2_ function as micrometer-scale messengers under relay-controlled ‘eustress’, reversibly fine-tuning immune activation via specific cysteine oxidation. At pathological levels, overt accumulation of either radicals or non-radicals disrupts immune homeostasis. While radicals cause widespread macromolecular damage and hyperinflammation, excessive non-radicals overwhelm redox relays, inducing irreversible protein oxidation and, ultimately, chronic inflammation. **c** For metabolism regulation, at physiological levels, radical species like •O_2_^−^ act within nanoscale domains, directly modifying iron-sulfur clusters and enzymes to rapidly rewire metabolic flux, e.g., redirecting the metabolic flux away from oxidative phosphorylation to favor glycolysis by inactivating iron-sulfur cluster-containing enzymes in the TCA cycle, enabling acute metabolic adaptation under stress. In contrast, non-radical H_2_O_2_ operates over micrometer scales, reversibly oxidizing signaling proteins like PTP1B via PRX-Trx relays to fine-tune glucose metabolism by, e.g., shifting the glycolytic flux toward the pentose phosphate pathway, while also modulating hypoxia by stabilizing HIF-1α. At pathological levels, excessive radical or non-radical ROS both irreversibly oxidize metabolic sensors, leading to disrupted mitohormesis. Light and dark intensities of each color represent low and high concentrations of radical or non-radical ROS, respectively. Additional abbreviations: AMPK, AMP-activated protein kinase; H_2_O_2_, hydrogen peroxide; HIF-1α, hypoxia-inducible factor 1 alpha; MAPK, mitogen-activated protein kinase; OXPHOS, oxidative phosphorylation; PPP, pentose phosphate pathway; PRX, peroxiredoxin; PTP, protein tyrosine phosphatase; PTP1B, protein tyrosine phosphatase 1B; ROS, reactive oxygen species; TCA, tricarboxylic acid cycle; Trx, thioredoxin; •O_2_^−^, superoxide anion

At low physiological concentrations, radical ROS such as •O_2_^−^ boost cell growth and proliferation through mitogenic redox signaling. This stimulatory effect is mediated by the precise and reversible oxidation of specific cysteine residues in key regulatory proteins, particularly protein tyrosine phosphatases (PTPs) (Fig. 4a). By transiently inhibiting PTPs, low-dose •O_2_^−^ activates growth-promoting pathways, such as the mitogen-activated protein kinase (MAPK) axis, thereby creating an intracellular environment conducive to DNA synthesis and cell cycle progression [73, 74]. Additionally, these radicals activate transcription factors like nuclear factor κB (NF-κB) and NRF2, which upregulate genes that support proliferation and enhance cellular antioxidant defenses, consolidating a pro-growth adaptive response. Thus, within a narrow low-concentration window, radical ROS function as crucial secondary messengers that foster survival and growth.

In stark contrast, a high dose of radical ROS triggers regulated cell death, with ferroptosis being a central pathway driven by radical lipid peroxidation (Fig. 4a). As redox flux increases, radical ROS not only downregulate growth receptors but directly initiate macromolecular damage. Specifically, radical-driven lipid peroxidation of cellular membranes acts as a critical execution signal [75, 76]. In ferroptosis, iron-dependent propagation of phospholipid peroxides overwhelms the primary defense enzyme GPX4 [76, 77], and peroxidized lipid bilayers can activate mechanosensitive channels like Piezo1, linking oxidative damage to lethal calcium influx [78]. The cellular threshold for this fate is determined by a network of parallel antioxidant systems, including the FSP1-CoQ_10_ axis that quenches lipid radicals at the membrane [79], and mitochondrial DHODH which regenerates the antioxidant coenzyme QH_2_ to suppress mitochondrial-associated ferroptosis [80]. The collaboration of pathways like DHODH and mitochondrial GPX4 in specific tissues, such as in neuroprotection post-injury [81], highlights the organized defense against high-dose radical stress, underscoring that pathological radical ROS levels pivot cell fate from growth to irreversible death.

#### Radical ROS in cell metabolism: plasticity vs. dysfunction

At a low physiological level, radical ROS such as •O_2_^−^ and •OH act as direct, rapid, and spatially restricted metabolic regulators that boost cellular adaptation. Their primary mechanism involves site-specific modifications of critical metabolic nodes, most notably the reversible inactivation of labile iron-sulfur clusters in enzymes like mitochondrial aconitase and electron transport chain complexes [82–85]. This targeted redox signal serves as an early alarm, acutely rewiring metabolic flux, for instance, away from the TCA cycle and towards alternative pathways like glycolysis or glutaminolysis, to meet changing energetic or biosynthetic demands, particularly under stress conditions such as inflammation or ischemia–reperfusion [86]. Further, these radicals activate key stress-response pathways, including AMPK and p53, and redox-sensitive transcription factors like HIF-1α, collectively promoting a catabolic, adaptive state [87–90]. Thus, within confined subcellular domains, low-dose radical ROS flux functions as a precise rheostat for metabolic plasticity, enabling rapid survival responses (Fig. 4b).

However, at a high, sustained dose, radical ROS trigger a cascade of irreversible damage that leads to metabolic dysfunction and collapse. Excessive •O_2_^−^ irreversibly oxidizes and degrades iron-sulfur clusters in core metabolic enzymes, such as aconitase and site I_Q_, disrupting ATP synthesis and anabolic precursor supply [88]. This direct enzyme damage is compounded by the inactivation of crucial detoxification systems, such as glyoxalase I (GLOI), leading to the toxic accumulation of methylglyoxal and advanced glycation end products (AGEs) that damage proteins and nucleic acids [91–93]. Furthermore, sustained radical stress inflicts widespread structural damage on organelles, most critically the mitochondria, through mechanisms like cardiolipin peroxidation (which disrupts cristae architecture) and mitochondrial DNA (mtDNA) mutations [94–97]. This cumulative damage impairs oxidative phosphorylation efficiency, collapses the cellular energy charge, and can integrate with cell death pathways such as ferroptosis [98–100]. Therefore, the transition from a low-dose signaling role to a high-dose damaging role represents a fundamental shift from adaptive metabolic rewiring to bioenergetic failure (Fig. 4b).

#### Radical ROS in immunity: homeostasis vs. pathological inflammation

At low, physiologically controlled doses, radical ROS such as •O_2_^−^ act as precise, spatially confined triggers that boost immune defense and regulation. Due to their short diffusion lengths and rapid scavenging, these radicals exert their effects within nanometer-to-submicrometer microdomains, making receptor-proximal membranes and specific enzyme complexes privileged initial targets [101–103] (Fig. 4c). This spatial constraint enables precise signaling, exemplified by the NADPH oxidase 2 (NOX2) complex, which assembles at receptor sites in antigen-presenting cells (APCs) and phagocytes. The locally generated •O_2_^−^ initiates near-field reactions and is rapidly converted by SODs to H_2_O_2_ [16–18, 101]. This H_2_O_2_ then functions as a diffusible secondary messenger, modulating key signaling nodes like protein tyrosine phosphatases to regulate immune cell communication and activation thresholds [16–18, 101]. Similarly, low-level mitochondrial •O_2_^−^ production, confined to its site of origin and converted to H_2_O_2_, contributes to immunometabolic adaptations such as the shift to aerobic glycolysis in activated immune cells, supporting effector functions like cytokine production [104]. Thus, tightly buffered radical ROS flux serves as a critical mechanism for activating and fine-tuning the immune response.

In contrast, a high, pathological dose of radical ROS, resulting from sustained NOX activation, mitochondrial dysfunction, or antioxidant failure, triggers destructive inflammation and disrupts immune homeostasis. The loss of spatial and quantitative control leads to uncontrolled •O_2_^−^ and •OH production that overwhelms local containment mechanisms. This results in the widespread, non-specific oxidation of host macromolecules, including lipids, proteins, and nucleic acids, which directly exacerbates tissue injury in conditions like acute lung injury and sepsis. Crucially, the loss of spatial restriction causes collateral oxidation of extracellular signaling receptors and adhesion molecules, dysregulating immune cell activation and promoting hyperinflammatory responses [17, 18, 87, 105, 106]. Furthermore, persistent radical stress induces maladaptive immunometabolic shifts, such as impaired oxidative phosphorylation and defective autophagy, which compromise the resolution of inflammation and contribute to the establishment of chronic inflammatory and autoimmune states. Therefore, the transition from a low-dose signaling role to a high-dose damaging role marks the shift from controlled immune defense to pathological inflammation.

### Physiological roles of non-radical ROS in cellular signaling and homeostasis

Non-radical ROS, e.g., H_2_O_2_, are fundamentally suited for their physiological roles in cellular signaling and homeostasis due to their unique chemical stability, membrane permeability, and capacity for enzymatic regulation. Their extended spatiotemporal range contrasts with the confined, near-field activity of radical ROS, allowing them to serve as diffusible, long-range messengers that integrate redox information across cellular compartments [5, 8]. This signaling capacity is executed through a structured ‘reach, deliver, read, reset’ cycle. The cycle is initiated by peroxiporins (e.g., AQP3) that facilitate H_2_O_2_ transmembrane flux to shape organelle-cytosol concentration gradients [8, 107]. The signal is then ‘delivered’ via high-fidelity relay systems, primarily the PRX-Trx axis, which transfers oxidizing equivalents with high specificity to client proteins such as PTPs and transcription factors like signal transducer and activator of transcription 3 (STAT3) [49, 108, 109]. At the ‘read’ step, reversible oxidation of key cysteine residues in these targets (e.g., transient PTP inhibition) encodes signal magnitude and duration, modulating kinase-driven pathways for adaptation [2, 9, 110]. Finally, the system is ‘reset’ through reduction by Trx and transcriptional programs like the Keap1-NRF2 axis, which restores redox homeostasis and elevates cytoprotective gene expression [10, 111]. This entire process is underpinned by a tiered enzymatic buffering system (PRXs, GPx, CAT) that maintains H_2_O_2_ in the nanomolar range, sculpts its subcellular gradients, and protects the reversibility of thiol switches, thereby preventing indiscriminate oxidation [5, 16–18, 31]. Consequently, physiological H_2_O_2_ flux facilitates oxidative eustress, enabling precise regulation of growth, metabolism, and immune function. In contrast, pathological overproduction saturates these relay and buffering systems, leading to irreversible oxidative damage and, consequently, cell death, metabolic dysfunction and pathological inflammation, highlighting a dose-dependent duality central to cellular redox biology (Fig. 3).

#### Non-radical ROS in cell fate: survival vs. death

At low physiological concentrations, H_2_O_2_ functions as a non-radical second messenger that boosts cell growth and adaptive responses [5] (Fig. 4a). This role is mediated through structured, reversible signaling circuits, primarily regulated by the PRX-Trx relay system. This system buffers and directs low-flux H_2_O_2_ to specific target proteins, leading to selective cysteine oxidation [112]. For example, H_2_O_2_ reversibly inhibits key PTPs, facilitating the activation of growth-factor kinase cascades such as MAPK and p38 [2, 113]. Simultaneously, it activates energy-sensing and stress-response pathways; H_2_O_2_ oxidizes AMPK to promote fatty-acid oxidation and redox balance, and it activates ATM kinase to link oxidative cues to AMPK-p53 signaling for survival [87, 114, 115]. Live-cell imaging confirms that these pulses induce temporally ordered, rather than globally damaging, transcriptional programs, enabling precise signal encoding for cell-cycle progression, migration, and survival [116]. Thus, low-dose non-radical ROS act as critical regulators of pro-growth adaptation.

Conversely, at high concentrations, H_2_O_2_ triggers cell death, primarily through cell cycle arrest and apoptosis, by overwhelming the protective buffering capacity of redox relays (Fig. 4a). When H_2_O_2_ levels increase, the PRX-Trx system becomes saturated and hyperoxidized, creating a sharp, ultrasensitive switch from adaptive signaling to oxidative damage [117]. This transition halts cell growth by inducing arrest at specific cell cycle checkpoints, such as the G1 phase, as demonstrated in lung cancer cells [118, 119]. Furthermore, sustained high-flux H_2_O_2_ drives apoptotic pathways; for instance, it mediates the cytotoxic effects of agents like nitro-aspirin in metastatic prostate cancer cells [120]. This dose-dependent logic illustrates a fundamental dichotomy: diffusible, low-flux non-radical signals promote adaptation and growth, while excessive non-radical ROS flux acts as a potent inducer of growth arrest and programmed cell death.

#### Non-radical ROS in cell metabolism: plasticity vs. dysfunction

At a low physiological dose, non-radical ROS, primarily H_2_O_2_, boost metabolic adaptation and plasticity by acting as reversible, enzyme-specific signaling molecules (Fig. 4b). This function is mediated through structured redox relays, where the PRX-Trx system captures H_2_O_2_ and transfers oxidizing equivalents to specific target proteins [5, 18, 40, 70, 121]. This results in the precise, reversible oxidation of key regulatory nodes, exemplified by the transient inhibition of protein tyrosine phosphatase 1B (PTP1B), which allows for the activation of kinase-driven pathways like insulin signaling [122]. Tissue-level circuits, such as NOX4-derived paracrine H_2_O_2_ enhancing skeletal muscle glucose uptake, directly connect this redox signaling to systemic metabolic control [123, 124]. Furthermore, low-dose H_2_O_2_ promotes a metabolic anticipatory response by redirecting glycolytic flux into the pentose phosphate pathway (PPP) to generate NADPH, bolstering cellular reductive capacity and resilience [125–129]. It also stabilizes transcription factors like HIF-1α under hypoxia, reprogramming glycolysis and angiogenesis to support adaptation [130–132]. Thus, controlled non-radical ROS flux fine-tunes energy and biosynthetic pathway selection for metabolic homeostasis.

In stark contrast, a high, pathological dose of non-radical ROS triggers metabolic dysfunction by overwhelming these protective relay and buffering systems (Fig. 4b). Excessive H_2_O_2_ flux saturates the PRX-Trx antioxidant capacity, leading to irreversible oxidation and permanent inactivation of critical metabolic sensors and enzymes, such as PTP1B and key dehydrogenases [133–135]. This disrupts insulin signaling, promotes insulin resistance, and inhibits mitochondrial function, shifting redox signaling from adaptive to detrimental [136, 137]. Sustained high fluxes drive a cascade of pathological outcomes, including oxidative stress, ER stress, lipotoxicity, and apoptosis in metabolic tissues like the liver, adipose tissue, and muscle [138–142]. Consequently, the loss of spatiotemporal control over non-radical ROS transforms them from homeostatic regulators into central drivers of metabolic disease progression [137, 143].

#### Non-radical ROS in immunity: homeostasis vs. pathological inflammation

At a low, physiologically controlled dose, non-radical ROS, primarily H_2_O_2_, function as essential and precise signaling molecules that boost immune defense and homeostasis (Fig. 4c). Low-flux H_2_O_2_ is captured and buffered by PRXs and relayed via the Trx system to specific target proteins, such as kinases and phosphatases, resulting in the reversible oxidation of key cysteine residues [112]. This relay-mediated eustress fine-tunes critical immune processes, including leukocyte activation thresholds and the transcriptional programs of inflammatory resolution, without causing collateral damage [109, 131, 144]. Furthermore, within the spatially confined environment of the phagosome, H_2_O_2_ serves as a precursor for potent antimicrobial agents. Myeloperoxidase (MPO) converts it to HOCl, which efficiently chlorinates and destroys ingested microbes [145], while its reaction with •NO forms ONOO^−^, contributing to microbial killing through nitration [146, 147]. Thus, low-dose non-radical ROS act as regulated secondary messengers and direct effectors for host defense.

In stark contrast, a high, pathological dose of non-radical ROS triggers extensive inflammation and tissue damage by overwhelming endogenous containment systems (Fig. 4c). Excessive H_2_O_2_ flux saturates the PRX-Trx buffering capacity, leading to irreversible oxidation of signaling proteins and disruption of normal immune regulation [12, 105, 148]. The antimicrobial MPO-HOCl system becomes a source of collateral damage; dysregulated HOCl production is linked to host tissue injury and complications like sepsis-induced atrial fibrillation [149]. Similarly, excessive ONOO^−^ formation causes widespread nitration of host proteins (e.g., forming 3-nitrotyrosine), altering their function and driving pathology in chronic inflammatory and autoimmune diseases [13, 150]. MPO overactivity is particularly implicated in exacerbating tissue injury and dysregulating cell death pathways such as NETosis [151]. Consequently, the loss of spatiotemporal control over non-radical ROS transforms them from homeostatic regulators to hallmarks of inflammatory disease and sources of pathological biomarkers.

## ROS in disease pathogenesis

Both radical and non-radical ROS exhibit dual and often paradoxical roles in the pathogenesis of major human diseases (Tables 1 and 2). Their biological impact is not inherently good or evil but is fundamentally determined by a critical interplay of dose, spatiotemporal context, and the integrity of cellular redox-buffering systems. Radical ROS, acting as short-range, highly reactive cytotoxic agents, drive disease through mechanisms like direct genomic damage and self-amplifying lipid peroxidation, linking them to cancer, neurodegeneration, and the cumulative tissue dysfunction of aging. In parallel, non-radical ROS, functioning as more stable, diffusible signaling molecules, can be therapeutically harnessed to combat infection and promote regeneration or, conversely, subverted to foster pathological processes like chronic inflammation, tumor progression, and age-related redox dyshomeostasis under abnormal flux regulation. By comparing and contrasting these two classes of oxidants across different disease etiologies, the following analysis presents a unified framework for understanding ROS in disease pathogenesis, where the transition from health to pathology represents a breakdown in the precise control of redox signaling rather than a simple overabundance of oxidants.

**Table 1:** Exemplified clinical studies of ROS-targeting therapeutics in this paper

ROS type	Primary ROS	Function	Modality	Medication context	Mechanism	Evidence level	Key endpoint	Ref(s)
Radical	•O_2_^−^ generation	Anti-oxidant	Oral vitamin E (400 IU every other day) + vitamin C (500 mg/day) supplementation	Primary prevention of cardiovascular disease in middle-aged and older men	Non-selective antioxidant supplementation → suppression of LDL-associated lipid peroxidation signaling → attenuation of redox-driven atherogenic pathways	Large-scale double-blind RCT (*n* = 14,641; mean follow-up 8 years)	No reduction in major cardiovascular events (myocardial infarction, stroke, or cardiovascular death) with vitamin E or C supplementation	[152]
Radical	•O_2_^−^ generation	Anti-oxidant	Oral vitamin E 400 IU daily	High-risk atherosclerotic cardiovascular disease	Chronic antioxidant exposure → reduced LDL oxidative modification → blunting of redox-dependent atherogenic signaling	Double-blind RCT (*n* = 9,541; mean follow-up 4.5 years)	No benefit on composite myocardial infarction, stroke, or cardiovascular death: 16.2% vs 15.5%; relative risk 1.05; *P* = 0.33	[153]
Radical	•O_2_^−^ neutralization	Anti-oxidant	Oral NAC, 1.2 g/day for 30 days	Exercise performance in adults with low baseline glutathione	NAC supplementation → expansion of glutathione-dependent redox buffering capacity	Placebo-controlled crossover RCT (*n* = 36; NCT1530646)	Only the low-glutathione subgroup improved performance: maximal oxygen uptake + 13.6%; highest achieved cycling power + 7.1%; time-trial performance + 15%. Oxidative-stress markers decreased mainly in low/moderate subgroups: plasma F2-isoprostanes −22% (low) and −14% (moderate); protein carbonyls −18% (low). High-glutathione subgroup showed no benefit and time-trial −3.5%	[154]
Radical	•O_2_^−^ neutralization	Anti-oxidant	Oral beta carotene 30 mg/day + retinyl palmitate 25,000 IU/day	Primary prevention from lung cancer for smokers and asbestos-exposed workers	High-dose β-carotene → neutralize endogenous radicals	Multicenter double-blind placebo-controlled RCT (*n* = 18,314; mean follow-up 4.0 years)	Terminated due to increased cancer incidence. Higher lung cancer incidence with supplements: relative risk 1.28; increased all-cause mortality: relative risk 1.17; deaths from lung cancer relative risk 1.46; cardiovascular death relative risk 1.26; trial stopped early for harm signal	[155]
Radical	•O_2_^−^ generation by exercise; •O_2_^−^neutralization by vitamins	Anti-oxidant	Vitamin C + vitamin E supplementation + endurance training	Exercise training adaptation in healthy adults	Antioxidant supplementation → suppression of exercise-induced ROS signaling → blunted redox-sensitive mitochondrial biogenesis	Double-blind RCT (*n* = 54; 11-week duration)	Training improved maximal oxygen uptake and running performance similarly in both groups, but the endurance training-induced increase in COX4 protein and cytosolic PGC-1α was blunted by anti-oxidant supplementation	[156]
Radical	•O_2_^−^ generation by exercise; •O_2_^−^neutralization by vitamins	Anti-oxidant	Vitamin C 1000 mg/day + vitamin E 400 IU/day + a 4-week supervised exercise program	Exercise training adaptation in healthy young men	Antioxidant supplementation → inhibition of ROS-dependent metabolic signaling → failure of exercise-induced insulin-sensitizing adaptation	Randomized double-blind placebo-controlled RCT (*n* = 40 enrolled; *n* = 39 completed; NCT00638560)	Exercise increased insulin sensitivity only without antioxidants, shown by higher glucose uptake during a hyperinsulinemic euglycemic clamp and higher adiponectin; these improvements were absent with vitamin supplementation	[157]
Radical	CAP (•O_2_^−^)	Anti-oxidant	CAP	Cutaneous microcirculatory dysfunction	CAP → low-level •O_2_^−^ generation → redox-sensitive vascular signaling → vasodilation → improved cutaneous microcirculation without oxidative injury	Clinical case study	Sustained ↑ skin oxygen saturation (~ 8%), ↑ hemoglobin index (~ 14%), ↑ perfusion up to 60 min, effects confined to treated area	[158]
Radical	•OH and •O_2_^−^ generation	Pro-oxidant	FMISO PET/CT-guided IMRT boost to the hypoxic subvolume (77 Gy/35 fractions) + standard chemoradiotherapy	Locally advanced stage III-IV head-and-neck cancer treated with definitive radiochemotherapy	Tumor hypoxia → reduced ROS fixation → radioresistance; hypoxia-targeted dose boosting restores efficacy	Randomized Phase II (*n* = 53; NCT02352792)	5-year local tumor control 100% in non-hypoxic tumors vs 59% with standard dose in hypoxic tumors vs 84% with hypoxia-subvolume boost; hypoxic vs non-hypoxic *P* = 0.039; boost vs standard *P* = 0.150; overall toxicity largely similar between arms	[159]
Non-radical	H_2_O_2_ neutralization	Anti-oxidant	Sleep (target 8.5 h)	Weight management/obesity risk in habitual short sleepers with overweight	Sleep extension → increased sleep duration → reduced energy intake	Single-center parallel-group RCT (*n* = 80; NCT02253368)	Sleep duration + 1.2 h/night vs control; energy intake −270 kcal/day vs control; no significant effect on total energy expenditure; weight change −0.87 kg vs control	[160]
Non-radical	HOCl generation	Pro-oxidant	HOCl + Standardized care	Wound	HOCl-mediated oxidative and chlorinating stress → rapid microbial macromolecule inactivation	Randomized RCT (*n* = 20; NCT04771819)	Non-inferior wound re-epithelialization at day 10; accelerated early re-epithelialization at day 4 (+ 14%); reduced bacterial burden measured as CFU	[161]
Non-radical	^1^O_2_ generation	Pro-oxidant	Scaling and root planing + Rose Bengal-activated light therapy	Periodontitis	Photosensitizer activation → ^1^O_2_ generation → oxidative disruption of bacterial cell-wall integrity	Single-blind RCT (*n* = 30; 3-month follow-up)	Lower gum inflammation score; shallower periodontal pockets (mm); better tooth-support attachment (mm); lower viable bacterial counts measured as CFU	[162]
Non-radical	^1^O_2_ generation	Pro-oxidant	Regenerative surgery + topical gaseous ozone, sessions on post-operative days 0/1/3/7	Stage III-IV grade C periodontitis	ICG-PDT activation → non-radical oxidative stress → broad-spectrum antimicrobial inactivation	Single-blind RCT (*n* = 48; NCT05447026)	Overall CAL improved similarly across groups; no overall difference in improvement	[163]
Non-radical	H_2_O_2_ generation	Pro-oxidant	Add-on IV high-dose vitamin C with carboplatin–paclitaxel	Advanced NSCLC	High-dose intravenous vitamin C → increased intratumoral H_2_O_2_ → oxidative DNA damage	Phase II, open-label, single-arm, Simon two-stage trial (*n* = 38; NCT02420314)	Objective tumor response 34.2%; disease control 84.2%; median PFS 5.7 months; median OS 12.8 months; severe treatment-related toxicity: 1 fatal neutropenic fever, 5 severe cytopenias	[164]
Non-radical	H_2_O_2_ generation	Pro-oxidant	Intravenous high-dose vitamin C + standard care	Glioblastoma	High-dose intravenous vitamin C → intratumoral H_2_O_2_ accumulation → oxidative stress constrained by tumor peroxide-detox capacity	Single-arm Phase II (*n* = 55; NCT02344355)	Iron-handling biomarkers correlated with outcomes: higher tumor transferrin receptor protein predicted shorter OS and PFS	[165]
Non-radical	H_2_O_2_ generation	Pro-oxidant	Intravenous high-dose vitamin C + gemcitabine + albumin-bound paclitaxel	Metastatic pancreatic ductal adenocarcinoma	High-dose intravenous vitamin C → H_2_O_2_ generation → secondary •OH formation → oxidative DNA damage	Randomized Phase II (*n* = 34; NCT02905578)	OS improved (16.2 vs 8.3 months); PFS improved (6.2 vs 3.9 months)	[166]
Non-radical	^1^O_2_ generation	Pro-oxidant	Topical 10% 5-aminolevulinic acid with either indoor daylight activation or red-lamp activation; lesion counts tracked at 1 and 6 months	Actinic keratosis	5-aminolevulinic acid-mediated photosensitization → ^1^O_2_ generation → localized oxidative cellular injury	Prospective clinical trial (*n* = 41; NCT03805737)	No significant difference in lesion clearance between red lamp and indoor daylight at 1 month and 6 months (clearance percentages reported for both arms)	[167]
Hybrid	CAP (H_2_O_2_, O_3_, •OH, ^1^O_2_, •O_2_^−^, etc.)	Anti-oxidant	CAP	Healthy human skin	CAP → local ROS generation → redox-mediated endothelial signaling → vasodilation → increased cutaneous microcirculation	Clinical case study	Significantly increased cutaneous microcirculation, reflected by enhanced local blood flow and tissue oxygenation following CAP exposure	[168]
Hybrid	CAP (H_2_O_2_, O_3_, •OH, ^1^O_2_, •O_2_^−^, etc.)	Pro-oxidant	Intraoperative CAP applied to surgical margins after tumor resection	Advanced (stage IV) or recurrent solid tumors	CAP → oxidative damage to DNA, proteins, and lipids at surgical margins	Phase I (*n* = 20; NCT04267575)	Safety demonstrated with no treatment-related adverse events and no clinically meaningful intraoperative physiologic perturbations; exploratory signals suggested improved local–regional control and survival/response stratified by surgical margin status	[169]
Hybrid	CAP (H_2_O_2_, O_3_, •OH, ^1^O_2_, •O_2_^−^, etc.)	Pro-oxidant	CAP + Standard care; 2 min once or twice weekly for up to 12 weeks	Chronic venous leg ulcers (slow-/non-healing) in primary care	CAP → oxidative damage to bacterial membranes and intracellular macromolecules	Randomized open-label RCT (*n* = 46; NCT04922463)	Complete wound closure within 12 weeks: 25.0% (standard care) vs 53.3% (CAP 1 ×/week) vs 61.5% (plasma 2 ×/week). Wound area reduction: 52.8% vs 63.9% vs 95.2%. Safety: no device-related serious adverse events	[170]
Hybrid	CAP (H_2_O_2_, O_3_, •OH, ^1^O_2_, •O_2_^−^, etc.)	Pro-oxidant	CAP + standard care	Wound	CAP → oxidative damage to microbial membranes and biofilm structures	Randomized placebo-controlled RCT (NCT04205942)	Wound area: mean remaining area 30.5% (CAP) vs 55.2% (placebo), *P* = 0.03; no CAP-related adverse events	[171]
Hybrid	CAP (H_2_O_2_, O_3_, •OH, ^1^O_2_, •O_2_^−^, etc.)	Pro-oxidant	CAP + standard care; 5 min/session, 3 times/week for 3 weeks	Diabetic foot-ulcer wound	CAP → oxidative damage to bacterial membranes and intracellular macromolecules	Double-blind RCT (*n* = 44; IRCT2008090401199N2)	Wound area improved (fractional wound size reduction, *P* = 0.02); at week 3, ≥ 50% wound-area reduction achieved in 77.3% vs 36.4% (CAP + standard care vs standard care)	[172]
Hybrid	CAP (H_2_O_2_, O_3_, •OH, ^1^O_2_, •O_2_^−^, etc.)	Pro-oxidant	CAP; 2 min per wart, up to 3 sessions, 4-week intervals	Pediatric viral warts	CAP → transient membrane permeabilization → oxidative modification of viral proteins and host-cell DNA	Randomized open-label RCT (*n* = 14; lesions = 112; NCT05070754)	Complete clearance: 51.8% CAP vs 60.7% cryotherapy; no significant difference. Pain was much lower with CAP; no serious adverse events	[173]
Hybrid	CAP (H_2_O_2_, O_3_, •OH, ^1^O_2_, •O_2_^−^, etc.)	Pro-oxidant	CAP; daily for 2 weeks	Malassezia folliculitis	CAP → oxidative disruption of fungal cell wall and membrane integrity	Randomized observer-blind active-controlled RCT (*n* = 50; 49 completed; NCT04886323)	Week-2 clinical success 40.0% CAP vs 58.3% itraconazole; microscopy negative 56.0% vs 66.7%; both improved quality-of-life; CAP had fewer adverse effects	[174]
Hybrid	CAP (H_2_O_2_, O_3_, •OH, ^1^O_2_, •O_2_^−^, etc.)	Pro-oxidant	CAP	Hyperseborrhea	CAP → transient ROS penetration into sebaceous units → suppression of sebocyte proliferation (Ki-67↓) + inhibition of lipogenesis (PPARγ↓)	Clinical case study	Reduced facial sebum levels and porphyrin index; decreased Ki-67⁺ sebocytes without thermal injury	[175]

*Abbreviations*: *CAL* clinical attachment level, *CAP* cold atmospheric plasma, *CFU* colony-forming unit, *COX4* cytochrome c oxidase subunit 4, *DNA* deoxyribonucleic acid, *FMISO* fluoromisonidazole, *GPX4* glutathione peroxidase 4, *Gy* gray, *H*_*2*_*O*_*2*_ hydrogen peroxide, *HOCl* hypochlorous acid, *ICG* indocyanine green, *ICG-PDT* indocyanine green–mediated photodynamic therapy, *IU* international unit, *Ki-67* marker of cellular proliferation Ki-67, *LDL* low-density lipoprotein, *NAC* n-acetylcysteine, *NCT* national clinical trial (ClinicalTrials.gov identifier), *NSCLC* non-small cell lung cancer, *OS* overall survival, *PET/CT* positron emission tomography/computed tomography, *PDT* photodynamic therapy, *PFS* progression-free survival, *PGC-1α* peroxisome proliferator-activated receptor gamma coactivator 1 alpha, *PPARγ* peroxisome proliferator-activated receptor gamma, *RCT* randomized controlled trial, *RNS* reactive nitrogen species, *ROS* reactive oxygen species, *SOD2* superoxide dismutase 2, ^*1*^*O*_*2*_ singlet oxygen, *•O*_*2*_^*−*^ superoxide anion, *•OH* hydroxyl radical, *O*_*3*_ ozone

**Table 2:** Exemplified preclinical studies of ROS-targeting strategies in this paper

ROS type	Leading ROS	Function	Modality	Medication context	Mechanism	Evidence level	Main in vivo outcome	Ref(s)
Radical	•O_2_^−^	Anti-oxidant	Exercise-preconditioning	Pressure-overload–induced cardiac hypertrophy/heart failure	Exercise-induced ROS → NRF2 activation → HO-1/NQO1/SOD2/GPX4 ↑ → oxidative stress ↓ → attenuation of hypertrophy and fibrosis	In vivo (mouse)	Exercise preconditioning reduced myocardial ROS, MDA accumulation, and preserved cardiac function and survival after TAC	[176]
Radical	•O_2_^−^	Anti-oxidant	Diet modulation	Neuronal-intestinal communication	•O_2_^−^ → dismutation by superoxide dismutase-2 → mitochondrial H_2_O_2_ ↑ → PKC-1 cysteine sulfenylation → dense-core vesicle exocytosis → FLP-1 neuropeptide release → NPR-4 signaling in intestine → SKN-1/NRF2-dependent antioxidant gene activation	In vivo* (C. elegans)*	•O_2_^−^ → dismutation by superoxide dismutase-2 → mitochondrial H_2_O_2_ ↑ → PKC-1 cysteine sulfenylation → dense-core vesicle exocytosis → FLP-1 neuropeptide release → NPR-4 signaling in intestine → SKN-1/Nrf2-dependent antioxidant gene activation	[177]
Radical	•O_2_^−^	Anti-oxidant	Paraquat	Lifespan	Mitochondrial •O_2_^−^ ↑ → RAS-dependent ROS signaling → oxidation of RAS Cys118 → enhanced cellular turnover and repair → lifespan extension	In vivo (*C. elegans*)	Low-dose paraquat or genetic elevation of mitochondrial •O_2_^−^ significantly extended lifespan and reprogrammed ~ 50% of the transcriptome	[178]
Radical	•O_2_^−^	Anti-oxidant	HIIT	Acute exercise	HIIT → redox-sensitive transcriptional activation → mitochondrial and metabolic remodeling	In vivo (mouse)	After 6 weeks, maximal running capacity increased in wild-type mice but the improvement was blunted in NOX2-deficient mice; mitochondrial network remodeling markers rose mainly in wild-type	[179]
Radical	•OH	Anti-oxidant	CAP + spermidine	Triple negative breast cancer	PAM → intracellular ROS elevation in TNBC → growth inhibition; Spd (anti-oxidant) → ROS scavenging → FTO/FOXO1 axis activation → enhanced antioxidant capacity → cancer cell survival → antagonizing PAM cytotoxicity	In vivo (mouse)	Spd reversed PAM-induced ROS accumulation and growth suppression in TNBC cells, promoting cell survival and proliferation via redox buffering and cell-cycle modulation	[180]
Radical	•O_2_^−^	Anti-oxidant	Low-intensity CAP	Wrinkle	Low-dose CAP → NRF2/ARE activation → endogenous antioxidant buffering ↑ → ECM protein expression ↑ → dermal remodeling	In vivo (mouse)	After 4–8 weeks, low-dose CAP significantly reduced UV-induced wrinkles in hairless mice; dermal collagen density increased and epidermal thickness decreased, with sustained effects after treatment cessation	[181]
Radical	•OH	Anti-oxidant	CAP	Skeletal muscle injury/muscle regeneration	CAP → low-level ROS generation → redox-sensitive signaling activation (satellite cell Pax7 activation; Ki-67 elevation) → enhanced myoblast proliferation, migration, and differentiation (MyoD/Myogenin induction) → formation of new myofibers without biomaterial support	In vitro (human myoblasts) + In vivo (mouse)	CAP significantly accelerated muscle defect repair and promoted regeneration of functional muscle tissue without inducing tissue damage	[182]
Radical	•O_2_^−^	Anti-oxidant	CAP activated solution	Diet-induced obesity	CAP → mild ROS signaling → suppression of ER stress/UPR (PERK–eIF2α–CHOP) → downregulation of PPARγ/C-EBPα → inhibition of adipocyte differentiation and lipogenesis	In vivo (mouse)	Daily intraperitoneal CAP reduced body weight gain, eWAT/ingWAT mass, adipocyte size, and triglyceride accumulation in high-fat diet–fed C57BL/6 mice without affecting food intake	[183]
Radical	LOOH•	Pro-oxidant	Tubastatin A + radiotherapy	Breast cancer	Radiotherapy → ROS generation → lipid peroxidation ↑ → GPX4 detoxification → ferroptosis; Tubastatin A → GPX4 inhibition → lipid peroxide accumulation → ferroptosis amplification	In vivo (mouse)	Enhanced radiotherapy-induced lipid peroxidation and tumor growth suppression	[184]
Radical	LOOH•	Pro-oxidant	RSL3 + radiotherapy	Glioblastoma	GPX4 inhibition → lipid ROS accumulation → impaired DNA damage repair → EMT suppression → radiosensitization	In vitro (glioma cells)	RSL3 markedly enhanced radiosensitivity of glioma cells by increasing lipid peroxidation, suppressing DNA damage repair, and inhibiting EMT, resulting in reduced clonogenic survival and tumor growth	[185]
Radical	LOOH•	Pro-oxidant	BIBR1532 + radiotherapy	Non-small cell lung cancer	Radiotherapy → ROS burst → lipid peroxidation → GPX4 suppression → ferroptosis → mitochondrial DNA release → cGAS-STING activation	In vivo (mouse)	Enhanced tumor ferroptosis, increased CD8^+^ T-cell infiltration, and improved antitumor immune response	[186]
Radical	LOOH•	Pro-oxidant	4-HNE exposure	Colorectal cancer	4-HNE accumulation → DNA damage → GLI1 ↑ → ATM/RAD51 ↑ → cancer stem cell self-renewal & asymmetric division → tumor growth	In vivo (mouse)	Increased tumor growth and LGR5^+^ CSC expansion with enhanced self-renewal and asymmetric division	[187]
Radical	•OH	Pro-oxidant	Amyloid-β	Alzheimer’s disease	Amyloid-β accumulation → iron dyshomeostasis → GPX4 inhibition and glutathione depletion → uncontrolled lipid peroxidation → ferroptotic neuronal death → synaptic dysfunction and neurodegeneration	In vivo (mouse)	Enhanced neuronal vulnerability, lipid peroxidation accumulation, and ferroptosis-associated neurodegeneration	[188]
Radical	•O_2_^−^	Pro-oxidant	ERCC1-deficiency	Vascular aging/endothelial dysfunction	DNA repair deficiency → mitochondrial dysfunction → •O_2_^−^ ↑ → oxidative stress → endothelial dysfunction	In vivo (mouse)	Accelerated vascular aging with impaired endothelium-dependent vasodilation and increased oxidative damage	[189]
Radical	LOOH•	Pro-oxidant	GPX4 deficiency	Sarcopenia	LOOH• accumulation → LOOH•-derived carbonyl stress (4-HNE, MDA) → lysosome-dependent proteolysis → muscle fiber atrophy and weakness	In vivo (mouse)	Increased LOOH• with aging and disuse; GPx4 loss exacerbated muscle atrophy and weakness, while genetic or pharmacological suppression of LOOH• prevented sarcopenia	[190]
Radical	•OH	Pro-oxidant	Ultrasound-triggered O_2_ microbubbles + radiotherapy	Breast cancer	O_2_ microbubbles → transient tumor oxygenation → enhanced oxygen-dependent DNA radical fixation → radiosensitization Microbubble cavitation → vascular barrier disruption → permeability increase	In vivo (mouse)	Improved primary tumor growth control after radiotherapy; however, increased lung metastatic burden associated with ultrasound-induced vascular disruption	[191]
Radical	•OH	Pro-oxidant	Ultrasound-triggered O_2_ microbubbles + radiotherapy	Neck squamous cell carcinoma	O_2_ release → transient tumor oxygenation → enhanced ROS-mediated DNA damage fixation	In vivo (mouse)	Delayed tumor growth and prolonged survival compared with radiation alone	[192]
Radical	LOO•	Pro-oxidant	FLASH radiotherapy + irradiation	Breast cancer, lung cancer	FLASH irradiation → transient ROS burst → iron-catalyzed lipid peroxidation	In vivo (mouse)	Tumors: lipid peroxidation increased after both FLASH and conventional irradiation; Normal tissues: FLASH showed lower lipid peroxidation and less injury than conventional irradiation; dietary iron loading abolished the FLASH protection	[193]
Radical	•OH	Pro-oxidant	Radiotherapy	Colorectal cancer, glioblastoma	Radiotherapy-activated NBTXR3 → local energy deposition ↑ → ROS amplification → DNA damage + ER stress → ICD	In vivo (mouse)	Improved local tumor control and CD8^+^ T-cell infiltration; enhanced systemic antitumor immune response	[194]
Radical	•OH	Pro-oxidant	CAP	Triple negative breast cancer	CAP → •OH-driven lipid peroxidation → EGFR(Y1068) phosphorylation → TRIM25-Keap1/SIAH2-NRF2 axis + p38-NRF2 axis → GPX4 transcriptional & translational suppression → ferroptosis	In vivo (mouse)	CAP significantly reduced TNBC tumor growth; effect was enhanced by sorafenib and blunted by EGFR(Y1068F) mutation	[195]
Radical	•OH	Pro-oxidant	CAP + EGF	Triple negative breast cancer	CAP + EGF → EGFR Tyr992/1173 phosphorylation → PLCγ activation → intracellular ROS amplification above apoptotic threshold → mitochondrial ATP production suppression → apoptosis	In vivo (mouse)	Combined CAP + EGF markedly suppressed TNBC tumor growth; effect was weakened by EGFR inhibition, confirming EGFR–ROS–apoptosis dependency	[196]
Radical	•OH	Pro-oxidant	Intratumoral delivery of CAP	Triple negative breast cancer	CAP → ROS burst → DNA damage → apoptosis	In vivo (mouse)	Compared with no treatment, CAP improved 30-day survival and inhibited tumor growth; induced tumor apoptosis and increased E-cadherin staining; improved inflammatory/immune blood indices and partially rescued renal dysfunction markers	[197]
Radical	LOOH•	Pro-oxidant	CAP	NSCLC	CAP → intracellular ROS accumulation → mitochondrial superoxide increase + labile iron accumulation → lipid peroxidation → downregulation of FSP1 independent of GPX4 → ferroptosis	In vitro (human cancer cell lines) + In vivo (mouse)	CAP significantly suppressed tumor growth in xenograft mice, accompanied by decreased FSP1 expression and increased 4-HNE and PTGS2	[198]
Non-radical	H_2_O_2_	Anti-oxidant	Physiological wound healing	Acute epithelial wound	Wounding → Duox1 activation → H_2_O_2_ gradient formation → redox-dependent leukocyte chemotaxis	In vivo (zebrafish)	Rapid leukocyte recruitment to wound site via tissue-scale H_2_O_2_ gradient	[199]
Non-Radical	H_2_O_2_	Anti-oxidant	NADPH oxidase activation	Alzheimer’s disease	Acetylcholine → nAChRα7 → Ca^2+^ influx → NADPH oxidase–derived •O_2_^−^ → SOD3-mediated conversion to H_2_O_2_ → aquaporin-facilitated H_2_O_2_ entry into neurons → Prx2-Trx2-TrxR1 redox relay → redox-sensitive signaling supporting long-term memory	In vivo (Drosophila)	Astrocyte-derived H_2_O_2_ gradient in mushroom body α-lobes is required for long-term memory, where amyloid-β blocks H₂O₂ signaling and impairs memory	[200]
Non-radical	H_2_O_2_	Anti-oxidant	NADPH oxidase activation	Cutaneous sensory nerve injury and wound	Tissue injury → Duox1 activation → H_2_O_2_ production → diffusion into keratinocytes and sensory axons → cysteine sulfenylation → EGFR oxidation at Cys797 → MMP-13 induction → extracellular matrix remodeling → axon regeneration	In vivo (zebrafish)	H_2_O_2_ is essential for wound-induced cutaneous sensory axon regeneration	[201]
Non-radical	Low-dose H_2_O_2_	Anti-oxidant	Topical H_2_O_2_ + excisional wound	Acute cutaneous wound	Low-dose H_2_O_2_ → redox signaling → endothelial activation → angiogenesis → accelerated wound closure	In vivo (mouse)	Low-dose H_2_O_2_ enhanced angiogenesis and wound closure	[202]
Non-radical	H_2_O_2_	Anti-oxidant	Acute exercise	Acute exercise	Acute exercise → mitochondrial H_2_O_2_ signaling → activation of redox-sensitive transcription factors (p66Shc, FOXO3a) → induction of adaptive antioxidant programs	In vivo (mouse)	Mitochondrial H_2_O_2_ increases time-dependently (max around 120 min); p66Shc and FOXO3a increase in parallel and correlate with H_2_O_2_; catalase activity rises at 120–150 min vs 90 min; total SOD activity unchanged	[203]
Non-radical	H_2_O_2_	Anti-oxidant	αMSH	metabolic syndrome	αMSH → ROS reduction → restoration of SOD activity → inhibition of FOXo1 signaling → activation of Rictor/mTORC2 → apoptosis suppression	In vivo (mouse)	αMSH significantly reduced ROS levels, restored antioxidant capacity, and protected adipocytes from oxidative stress–induced apoptosis	[204]
Non-radical	H_2_O_2_	Anti-oxidant	Pharmacological ascorbate + chemotherapy	Pancreatic ductal adenocarcinoma	Pharmacological ascorbate → extracellular H_2_O_2_ generation → selective oxidative pressure → upregulation of H_2_O_2_-detoxifying systems → maintenance of redox homeostasis → ascorbate resistance	In vivo (mouse)	Ascorbate-resistant cancer cells exhibit increased invasiveness, enhanced metastatic colonization, and higher circulating tumor cell burden	[205]
Non-radical	H_2_O_2_	Anti-oxidant	CAP + UVB	Squamous cell carcinoma	CAP → activation of NRF2 → induction of ARE-dependent transcription → remodeling of epidermal redox signaling under UVB exposure	In vivo (mouse)	CAP controlled UVB-driven epidermal thickening and reduced histology features of SCC progression (follicular atrophy, dermal degeneration, keratin pearls); repeated CAP on non-UVB skin caused no pathological or pre-malignant changes	[206]
Non-radical	H_2_O_2_	Anti-oxidant	Low-intensity CAP	Wrinkle	Low-dose CAP → H_2_O_2_-mediated redox signaling → NRF2 activation → ↑ antioxidant enzymes + collagen remodeling → wrinkle reduction	In vivo (mouse)	Reduced wrinkle depth; improved dermal thickness; enhanced collagen organization	[181]
Non-radical	H_2_O_2_	Pro-oxidant	EAAT3/EAAC1 knockout + α-synuclein overexpression	Parkinson’s disease-related α-synucleinopathy	EAAT3 deficiency → neuronal cysteine uptake ↓ → glutathione depletion → chronic oxidative stress → lipid peroxidation (4-HNE) → covalent modification of α-synuclein → accelerated α-synuclein aggregation (pSer129-α-syn) → motor impairment	In vivo (mouse)	Increased α-synuclein aggregate burden in cortex, hippocampus, and substantia nigra; worsened motor performance compared with α-synuclein alone	[207]
Non-radical	High-dose H_2_O_2_	Pro-oxidant	Topical H_2_O_2_ + excisional wound	Acute cutaneous wound	High-dose H_2_O_2_ → excessive proteolysis (MMP-8 ↑) → impaired matrix deposition → delayed healing	In vivo (mouse)	High-dose H_2_O_2_ delayed early healing without increasing lipid or protein oxidative damage	[202]
Non-radical	H_2_O_2_	Pro-oxidant	Metabolic stress	Insulin resistance	Enhanced FOXO1 activity → reduced insulin signaling → enhanced chronic H_2_O_2_-associated oxidative stress → reduced PPARγ-dependent metabolic homeostasis	In vivo (mouse)	Reduced hepatic and skeletal muscle insulin sensitivity with elevated inflammatory cytokines under high-fat diet	[208]
Non-radical	H_2_O_2_	Pro-oxidant	Free fatty acids	Insulin resistance	Free fatty acids → H_2_O_2_ ↑ → FOXO1 downregulation → MnSOD and glutathione peroxidase ↓ → amplified oxidative stress → pro-inflammatory adipokine shift	In vitro (3T3-L1 adipocytes) + In vivo (db/db mice)	Increased ROS production, reduced FOXO1 protein levels, pro-inflammatory adipokine profile, and insulin-resistant adipocyte phenotype	[209]
Non-radical	H_2_O_2_	Pro-oxidant	Pharmacological ascorbate + chemotherapy	NSCLC	High-dose ascorbate → extracellular H_2_O_2_ accumulation → tumor-selective oxidative stress → DNA damage → impaired tumor cell viability	In vivo (mouse)	Reduced tumor growth and enhanced treatment sensitivity with acceptable safety	[210]
Non-radical	H_2_O_2_	Pro-oxidant	Ascorbate	Pancreatic ductal adenocarcinoma	Repeated pharmacologic ascorbate exposure → selection for enhanced H_2_O_2_ detoxification → redox metabolic reprogramming → acquisition of oxidative stress-resistant phenotype	In vivo (mouse)	Resistant cells show higher invasive potential, more aggressive tumor colonization, and higher circulating tumor cell abundance	[205]
Non-radical	H_2_O_2_	Pro-oxidant	Dry H_2_O_2_	Environmental microbial contamination	DHP-derived H_2_O_2_ → oxidative inactivation of airborne and surface-associated microorganisms → reduction of total colony-forming units	Real-world hospital environmental study	Significant reduction of airborne and surface microbial bioburden in occupied patient rooms without exceeding human safety thresholds	[211]
Non-radical	H_2_O_2_	Pro-oxidant	Dry H_2_O_2_	Environmental microbial contamination	H_2_O_2_ decomposition → •OH generation → oxidative damage to microbial proteins, lipids, and nucleic acids → microbial inactivation	Real-world hospital environmental study	Surface culture positivity reduced from 20.5% to 5.1%; airborne species reduced by 36.1% after disinfection	[212]
Non-radical	O_3_	Pro-oxidant	O_3_ water	Environmental microbial contamination	O_3_-mediated oxidation → microbial membrane and protein damage → ATP depletion → microbial inactivation	Real-world hospital environmental study	Ozonated water reduced ATP bioluminescence to < 100 RLU and achieved higher surface disinfection efficacy (98.1%) than 70% alcohol (86.5%)	[213]
Non-radical	^1^O_2_	Pro-oxidant	CAP	Breast cancer, colorectal cancer, glioblastoma	CAP → interaction of H_2_O_2_ with NO_2_^−^ → primary ^1^O_2_ generation → selective inactivation of membrane-associated CAT → survival of cell-derived H_2_O_2_ and ONOO^−^ → secondary ^1^O_2_ amplification → aquaporin-mediated H_2_O_2_ influx → glutathione depletion → reactivation of intercellular HOCl signaling → •OH-driven lipid peroxidation → apoptosis	In vitro (human cancer cell lines)	CAP selectively induced apoptosis in malignant cells but not in non-malignant cells by triggering tumor-cell-driven RONS amplification and lipid peroxidation	[214]
Non-radical	H_2_O_2_	Pro-oxidant	Intratumoral delivery of CAP	Peritoneal carcinomatosis	H_2_O_2_-rich oxidized saline → intracellular glutathione oxidation → ER stress activation (IRE1/ATF6/XBP1 axis) → apoptosis	In vivo (mouse)	Repeated intraperitoneal treatment reduced peritoneal tumor burden and increased apoptosis in tumor nodules; plasma-oxidized saline and H_2_O_2_-matched control showed similar efficacy	[215]
Non-radical	H_2_O_2_	Pro-oxidant	CAP	Glioblastoma	CAP → oxidative DNA damage accumulation → cell-cycle arrest → enhanced vulnerability to temozolomide-induced genotoxic stress	In vivo (mouse)	Intracranial glioblastoma model: CAP alone reduced tumor growth (~ 40%), whereas CAP combined with temozolomide nearly abolished tumor progression	[216]
Non-radical	H_2_O_2_	Pro-oxidant	Intratumoral delivery of CAP	Melanoma, oral tongue squamous-cell carcinoma, non-small cell lung cancer	CAP → disruption of tumor lipid metabolic pathways → attenuation of LPA-dependent growth signaling	In vivo (mouse)	Robust tumor growth suppression across multiple xenograft models, accompanied by reduced tumor cell proliferation	[217]
Non-radical	H_2_O_2_	Pro-oxidant	CAP + cisplatin	Platinum-resistant ovarian cancer	CAP → modulation of redox-sensitive PI3K/AKT → altered GSK3β activity → changes in β-catenin stability → EMT rewiring	In vivo (mouse)	CAP + low-dose cisplatin (2.5 mg/kg) produced the strongest tumor control (lowest bioluminescence, lowest Ki-67) and the least intestinal/peritoneal metastasis; low-dose cisplatin alone showed little effect; high-dose cisplatin (5 mg/kg) inhibited tumor growth but was associated with systemic toxicity signals (weight loss and adverse serum biochemistry)	[218]
Non-radical	H_2_O_2_	Pro-oxidant	Topical use of CAP	Chemotherapy-associated oral mucositis with oral candidiasis	CAP → oxidative disruption of Candida cell envelope → loss of fungal membrane integrity → impairment of intracellular viability-associated signaling	In vivo (mouse)	24 h: Candida-positive cultures reduced vs control in lung (1/9 vs 9/10), liver (0/9 vs 5/10), spleen (0/9 vs 5/10); 72 h: no organ recovery in treated group (0/7 across lung/liver/spleen/kidney) while control remained positive (e.g., lung 5/9). Blood cultures were negative	[219]
Non-radical	H_2_O_2_	Pro-oxidant	CAP	Pancreatic ductal adenocarcinoma	CAP → intracellular oxidative stress exceeding redox tolerance in pancreatic cancer cells → exposure of calreticulin (ecto-CRT) + release of ATP and HMGB1 + downregulation of CD47 → ICD	In vitro (human cancer cell lines)	CAP selectively induced ROS-dependent ICD in pancreatic cancer cells but not in stellate cells, leading to enhanced dendritic cell phagocytosis and maturation and a more immunostimulatory tumor microenvironment	[220]
Non-radical	H_2_O_2_	Pro-oxidant	CAP + anti-PD-1 immune checkpoint blockade	Melanoma (murine B16F10; human melanoma models)	CAP → supraphysiological ROS/RNS → intracellular oxidative distress → glutathione depletion + mitochondrial dysfunction → caspase-3/7–dependent apoptosis and induction of ICD → enhanced antigen presentation and reduced immune-checkpoint expression → sensitization to anti-PD-1 therapy	In vivo (mouse)	CAP + anti-PD-1 significantly reduced tumor growth and increased remission rates compared with either treatment alone, accompanied by increased intratumoral immune infiltration	[221]
Non-radical	H_2_O_2_	Pro-oxidant	Trident CAP	Chronic myeloid leukemia	Tri-CAP → exceptionally low-level extracellular ROS + pulsed intracellular ROS elevation → excessive ROS production + attenuated antioxidant defense (reduced glutathione/oxidized glutathione imbalance) → simultaneous blockade of redox deregulation, glycolysis, and AKT/mTOR/HIF-1α signaling → predominant caspase-dependent apoptosis (with minor ferroptosis/necrosis contribution)	In vivo (mouse)	Tri-CAP markedly reduced tumor formation and prolonged survival of tumor-bearing mice while sparing non-malignant cells	[222]
Non-radical	H_2_O_2_	Pro-oxidant	CAP	Triple negative breast cancer	PAM → extracellular H_2_O_2_ accumulation → aquaporin-facilitated ROS influx → intracellular ROS overload → redox crisis → inhibition of hyperactivated MAPK/JNK and NF-κB signaling → apoptosis	In vitro (human cancer cell lines) + In vivo (mouse)	CAP selectively suppressed TNBC tumor growth in mice with minimal effect on non-TNBC tumors and no observable systemic toxicity	[223]
Non-radical	H_2_O_2_	Pro-oxidant	CAP	Androgen receptor–independent prostate cancer	PAM → ↑ intracellular ROS (mainly H_2_O_2_/O_3_) → ROS threshold exceeded in cancer cells → G0/G1 cell cycle arrest + apoptosis → ↓ ERK/p38/NF-κB phosphorylation and ↑ JNK activation → suppressed proliferation and migration	In vitro (human cell lines)	Selective induction of G_0_/G_1_ arrest and apoptosis in prostate cancer cells without significant damage to normal prostate epithelial cells	[224]
Non-radical	H_2_O_2_	Pro-oxidant	CAP + auranofin	Glioblastoma	CAP → ROS/RNS accumulation → oxidative stress; auranofin (thioredoxin reductase inhibitor) → blockade of antioxidant defense → amplified ROS burden → mitochondrial dysfunction → mixed regulated cell death (apoptosis + ferroptosis-like features) → damage-associated molecular pattern release (ATP, calreticulin exposure) → ICD	In vivo (mouse) + In vitro (human cancer cell lines)	CAP and auranofin synergistically enhanced ROS-dependent tumor cell death and promoted immunogenic responses compared with either treatment alone	[225]

*Abbreviations*: ^*1*^*O*_*2*_ singlet oxygen, *4-HNE* 4-hydroxynonenal, *AKT* protein kinase B, *ARE* antioxidant response element, *ATF6* activating transcription factor 6, *ATM* ataxia telangiectasia mutated, *ATP* adenosine triphosphate, *α-MSH* alpha-melanocyte-stimulating hormone, *α-syn* alpha-synuclein, *BIBR1532* telomerase inhibitor BIBR1532, *CAP* cold atmospheric plasma, *CAT* catalase, *CD8*^+^ *T cell* CD8-positive T lymphocyte, *cGAS* cyclic *GMP–AMP* synthase, *CFU* colony-forming unit, *CHOP* C/EBP homologous protein, *C/EBPα* CCAAT/enhancer-binding protein alpha, *CSC* cancer stem cell, *Cys118* cysteine residue at position 118, *DAMPs* damage-associated molecular patterns, *DHP* dry hydrogen peroxide, *DNA* deoxyribonucleic acid, *Duox1* dual oxidase 1, *EAAT1* excitatory amino acid transporter 1, *EAAT3* excitatory amino acid transporter 3, *ECM* extracellular matrix, *ecto-CRT* ectopic calreticulin exposure, *EGF* epidermal growth factor, *EGFR* epidermal growth factor receptor, *eIF2α* eukaryotic initiation factor 2 alpha, *EMT* epithelial–mesenchymal transition, *ER* endoplasmic reticulum, *ERCC1* excision repair cross-complementation group 1, *ERK* extracellular signal-regulated kinase, *eWAT* epididymal white adipose tissue, *FLASH* ultra-high dose rate radiotherapy, *FLP-1* FMRFamide-like neuropeptide-1, *FOXO1* forkhead box O1, *FOXO3a* forkhead box O3a, *FSP1* ferroptosis suppressor protein 1, *FTO* fat mass and obesity-associated protein, *GPX4* glutathione peroxidase 4, *GSK3β* glycogen synthase kinase 3 beta, *H*_*2*_*O*_*2*_ hydrogen peroxide, *HIF-1α* hypoxia-inducible factor-1 alpha, *HIIT* high-intensity interval training, *HMGB1* high-mobility group box 1, *HO-1* heme oxygenase-1, *HOCl* hypochlorous acid, *ICD* immunogenic cell death, *ingWAT* inguinal white adipose tissue, *IRE1* inositol-requiring enzyme 1, *JNK* c-Jun N-terminal kinase, *Keap1* Kelch-like ECH-associated protein 1, *Ki-67* marker of cellular proliferation, *LGR5*^+^ leucine-rich repeat-containing G-protein-coupled receptor 5-positive, *LOO•* lipid peroxyl radical, *LOOH* lipid hydroperoxide, *LOOH•* lipid hydroperoxyl radical, *LPA* lysophosphatidic acid, *MAPK* mitogen-activated protein kinase, *MDA* malondialdehyde, *MnSOD* manganese superoxide dismutase, *MMP-8* matrix metalloproteinase-8, *MMP-13* matrix metalloproteinase-13, *mTOR* mechanistic target of rapamycin, *mTORC2* mechanistic target of rapamycin complex 2, *MyoD* myogenic differentiation 1, *NADPH* nicotinamide adenine dinucleotide phosphate, *NF-κB* nuclear factor-Κb, *NO₂⁻* nitrite, *NOX2* NADPH oxidase 2, *NPR-4* neuropeptide receptor 4, *NQO1* NAD(P)H quinone dehydrogenase 1, *NRF2* nuclear factor erythroid 2–related factor 2, *NSCLC* non-small-cell lung cancer, *nAChRα7* nicotinic acetylcholine receptor alpha-7 subunit, *O*_*2*_ molecular oxygen, *•O*_*2*_^*−*^ superoxide anion, *O₃* ozone, *PAM* plasma-activated medium, *PD* Parkinson’s disease, *PD-1* programmed cell death protein-1, *PERK* protein kinase RNA-like endoplasmic reticulum kinase, *PI3K* phosphoinositide 3-kinase, *PKC-1* protein kinase C-1, *PLCγ* phospholipase C gamma, *PPARγ* peroxisome proliferator-activated receptor gamma, *p38* p38 mitogen-activated protein kinase, *p66Shc* 66 kDa Src homology and collagen adaptor protein, *PTGS2* prostaglandin-endoperoxide synthase 2, *RAD51* RAD51 recombinase, *RAS* rat sarcoma proto-oncogene, *Rictor* rapamycin-insensitive companion of mTOR, *RLU* relative light unit, *RNS* reactive nitrogen species, *RONS* reactive oxygen and nitrogen species, *ROS* reactive oxygen species, *RSL3* RAS-selective lethal 3, *SCC* squamous cell carcinoma, *SI A H 2 (SIAH2)* seven in absentia homolog 2, *SKN-1* skinhead-1 (Caenorhabditis elegans NRF2 ortholog), *SOD* superoxide dismutase, *SOD2* superoxide dismutase 2, *SOD3* superoxide dismutase 3, *Spd* spermidine, *STING* stimulator of interferon genes, *TAC* transverse aortic constriction, *TNBC* triple-negative breast cancer, *TRIM25* tripartite motif-containing protein 25, *TrxR1* thioredoxin reductase 1, *Tubastatin A* selective histone deacetylase 6 inhibitor, *UPR* unfolded protein response, *UV* ultraviolet radiation, *UVB* ultraviolet B radiation, *XBP1* X-box binding protein 1, *•OH* hydroxyl radical

### Radical ROS in disease pathogenesis

#### Mechanisms of pathogenic signaling

**Fig. 5:**
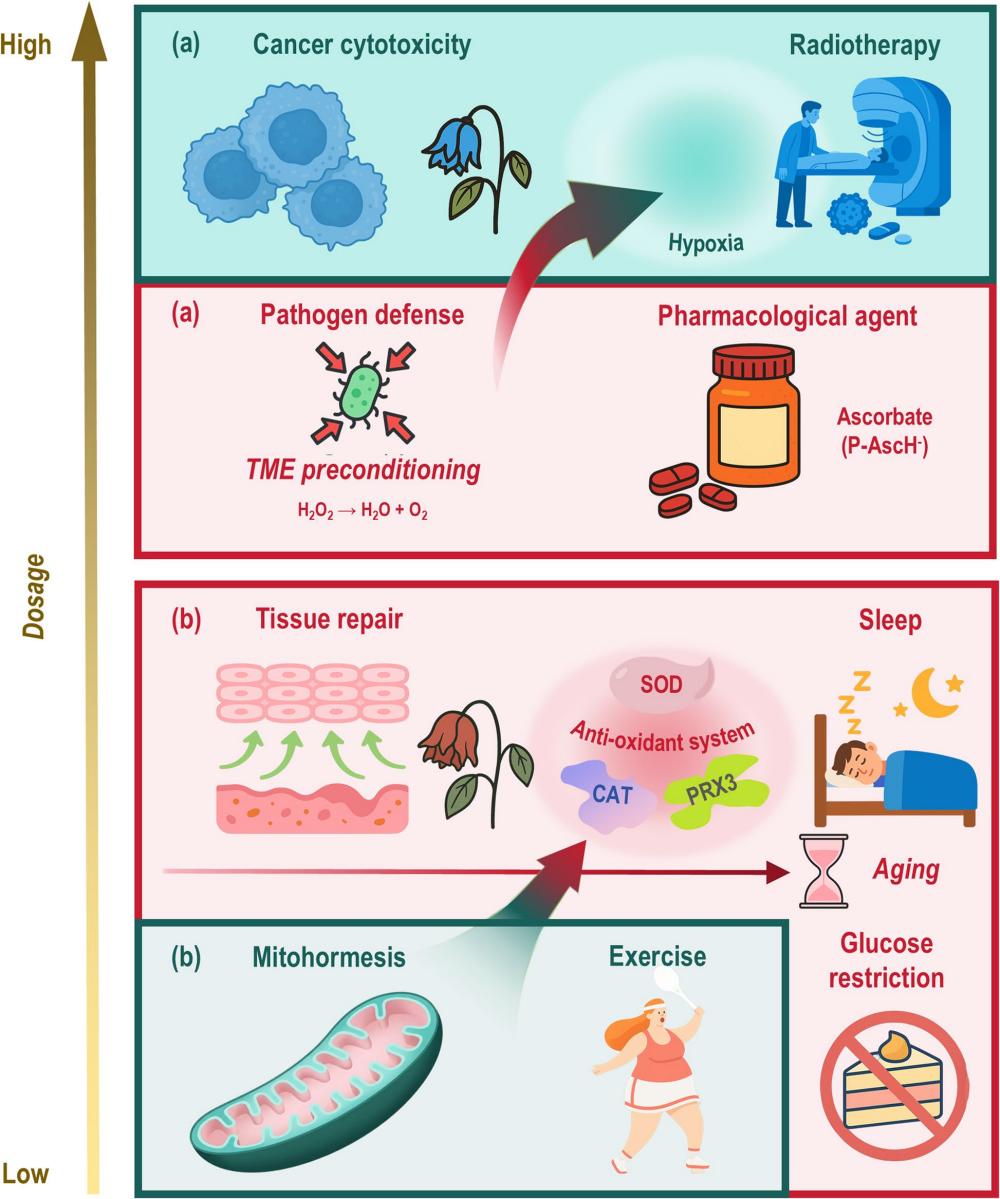
Radical versus non-radical ROS in disease pathogenesis. Radical and non-radical ROS play context-dependent roles in disease and health. In disease, radical-focused approaches such as radiotherapy induce localized, irreversible cytotoxicity via nanoscale reactions, the efficacy of which is, however, often limited by hypoxia (a in blue); whereas pharmacological agents generating none-radical ROS such as ascorbate (P-AscH^-^) induce programmable, spatially controlled effects that are crucial for antimicrobial defense and, importantly, tumor microenvironment preconditioning that can synergize with radicals for enhanced cancer cell killing (a in red) In health, physiological levels of non-radicals mediate tissue repair, the beneficial window of which, however, narrows with age-related mitohormesis decline (b in red); while interventions like physical exercise, sufficient sleep and glucose restriction can generate moderate levels of radicals to support mitohormesis (b in blue), thus, radicals and non-radicals can reinforce each other for daily health maintenance. Thereby, radical and non-radical ROS are leveraged in distinct therapeutic strategies based on their chemical properties that can reinforce each other once used in combination on appropriate dosage calibration. In summary, while radical therapies excel in rapid boost of mitohormesis and ablation of transformed cells, non-radical modalities offer tunable signaling, repeatable dosing, and broader applicability in tissue repair and tumor microenvironment preconditioning that can synergize with radicals for improved health and disease management. Blue and red represent radical and non-radical ROS, respectively. Respectively. Additional abbreviations: P-AscH^−^, pharmacological ascorbate; ROS, reactive oxygen species; RT, radiotherapy; TME, tumor microenvironment

Radical ROS drive disease pathogenesis by acting as highly reactive, short-range cytotoxic agents, primarily through two interconnected mechanisms, i.e., direct genomic damage and the initiation of self-amplifying lipid peroxidation cascades (Fig. 5a). The •OH, generated in close proximity to DNA via processes like water radiolysis during radiotherapy, reacts at near diffusion-limited rates to produce clustered DNA lesions. These lesions include mutagenic bases like 8-oxoguanine (8-oxoG) and double-strand break clusters, which are major drivers of genomic instability and cell death in well-oxygenated tumors [101, 226–228]. Alongside DNA damage, radicals such as •OH and lipid peroxyl radicals (ROO•) initiate and propagate lipid peroxidation chain reactions, particularly in membranes rich in polyunsaturated fatty acids (PUFAs). This process transforms a localized radical hit into widespread, irreversible membrane damage.

#### Disease associations

The relationship between radical ROS and disease, spanning, e.g., cancer, neurodegeneration, and aging, is fundamentally defined by a dose-dependent duality [176, 229], where physiological levels mediate essential adaptation but pathological accumulation drives irreversible macromolecular damage and functional decline (Fig. 5b). This manifests as genomic instability through the accumulation of mutagenic lesions like 8-oxoguanine (8-oxoG), which drives both disease risk and progression by acting as an epigenetic-like mark [230, 231].

In cancer, radical ROS play a paradoxical role. While low, localized fluxes can promote tumor cell survival and proliferation through redox signaling, therapeutic strategies deliberately exploit their high reactivity to induce cancer cell death. Radiotherapy, for instance, utilizes ionizing radiation to generate •OH via water radiolysis, causing clustered DNA double-strand breaks and lethal genomic instability in tumor cells [76, 77, 184, 185, 232]. Furthermore, the induction of ferroptosis, an iron-dependent, radical-driven cell death pathway centered on catastrophic lipid peroxidation, has become a promising therapeutic approach to eliminate therapy-resistant cancers, particularly those with compromised antioxidant defenses like downregulated GPX4 [76, 77, 184, 185, 232]. The cytotoxic potential of these radicals can be further amplified in combinatorial treatments, such as combining radiation with telomerase inhibitors to induce ferroptosis via the cyclic GMP-AMP synthase (cGAS)-stimulator of interferon genes (STING) pathway [185, 186]. Thus, the pathogenic signaling of radical ROS is rooted in their ability to cause concentrated, catastrophic damage to core cellular components. However, applying the lipid peroxidation agent 4-HNE promoted the stemness and progression of colorectal cancer cells by reinforcing cancer stem cell programs [187]. This paradoxical effect underscores that the biological outcome of radical-initiated damage is not uniformly cytotoxic but is critically shaped by the cellular and disease context.

In neurodegeneration, the brain’s high oxygen consumption, lipid-rich content, and relatively weak antioxidant defenses make it exceptionally vulnerable to radical ROS. The accumulation of oxidative damage to lipids, proteins, and mitochondrial DNA is a hallmark of diseases like Alzheimer’s and Parkinson’s [188, 207, 233]. Radical-initiated lipid peroxidation generates neurotoxic aldehydes such as 4-HNE, which form stable adducts with key neuronal proteins, disrupting function and promoting aggregation of pathological proteins like amyloid-β and α-synuclein.

The process of aging is intrinsically linked to the cumulative and systemic burden of radical ROS damage, which acts as a principal driver of functional decline across multiple tissues through two interconnected, self-reinforcing pathological axes, i.e., macromolecular damage and the erosion of regenerative capacity. The first axis involves direct, irreversible damage to core cellular components, causing clustered oxidative DNA lesions and reactive aldehydes, leading to a progressive disruption of metabolic homeostasis and the accumulation of dysfunctional cellular machinery [234, 235]. The second axis is the propagation of this damage into tissue-specific dysfunction. In the vasculature, oxidative damage to DNA and proteins is implicated in extracellular matrix remodeling and arterial stiffening, a key contributor to cardiovascular aging [189]. In skeletal muscle, elevated lipid peroxides and a decline in the key antioxidant enzyme GPX4 create a milieu permissive for ferroptosis-like pathways, driving the loss of muscle mass and function characteristic of sarcopenia; this process is experimentally mitigated by ferroptosis inhibitors like liproxstatin-1, directly linking radical chemistry to age-related atrophy [190]. Conversely, it is crucial to note that low-dose radical flux supports physiological adaptive homeostasis, e.g., in exercise preconditioning and mitochondrial hormesis. These findings underscore that the pathogenesis of diseases associated with radical ROS arises not from their mere presence, but from a decisive shift toward the irreversible damage axis when sustained high fluxes overwhelm cellular antioxidant and repair capacities, turning these essential molecules into drivers of degenerative pathology.

### Non-radical ROS in disease pathogenesis

#### Mechanisms of pathogenic signaling

The roles of non-radical ROS in disease pathogenesis are defined not only by their indiscriminate oxidative power, but more importantly by the precise mimicry or supplementation of their natural, low-flux physiological signaling pathways to orchestrate divergent cellular responses, yielding therapeutic benefit in some contexts while driving pathology in others (Fig. 5a).

In oncology, this principle is leveraged to counteract tumor immunosuppression and hypoxia. By blocking hypoxia-primed proliferation via reversible cysteine oxidation on proximal receptors, the controlled, intratumoral enzymatic generation of H_2_O_2_ (via lactate oxidase or glucose oxidase) can reoxygenate hypoxic areas, activate dendritic cells, and restore therapy sensitivity. For instance, calibrated intratumoral H_2_O_2_ combined with radiotherapy activated dendritic-cell (DC)/T-cell responses and elicited an abscopal effect, enhanced by PD-1 blockade [236]. Direct injection of formulations containing 0.5% H_2_O_2_ radiosensitized tumors by reoxygenation [237], while engineered systems like lactate oxidase nanocapsules converted immunosuppressive lactate into immunostimulatory H_2_O_2_, sensitizing tumors to anti-PD-1 therapy [238]. Similarly, tumor-targeted glucose oxidase generated H_2_O_2_ in situ, enhancing DC cross-presentation and inducing cancer cell death through pyroptosis or disulfidptosis when combined with immunotherapies [239, 240]. However, chronic, pathological levels of non-radical ROS in the tumor microenvironment conversely fostered immunosuppression and therapy resistance, highlighting their starkly context-dependent impact [3, 241].

In infection and immunity, non-radical ROS function as central effector molecules for host defense but can also contribute to tissue damage. Myeloperoxidase (MPO) converts H_2_O_2_ into potent HOCl within phagosomes for pathogen killing [12, 148, 242], while the DUOX-lactoperoxidase system at mucosal surfaces uses H_2_O_2_ to generate milder oxidants like hypothiocyanite (OSCN⁻), providing broad-spectrum antiviral and antibacterial defense that maintains tissue homeostasis [243–245]. The critical role of this pathway is confirmed in vivo, as Duox1 deficiency increases influenza mortality, and human airway epithelia-generated OSCN^−^ directly inactivates influenza strains [246]. Thus, the therapeutic or pathological outcome of non-radical ROS is exquisitely determined by their source, flux, duration, and the integrity of the local redox-buffering environment.

#### Disease associations

Non-radical ROS have been profoundly implicated in a large spectrum of etiologies encompassing, e.g., cancers, neurodegeneration, aging, tissue regeneration and infection.

In cancers, non-radical ROS act through dual mechanisms that either suppress or promote tumor progression depending on the context, concentration, and location within the tumor microenvironment (TME) (Fig. 5b). At controlled, low levels, non-radical ROS, primarily H_2_O_2_, can exert antitumor effects by functioning as signaling molecules that modulate immune responses and sensitize cancer cells to therapy. For instance, calibrated intratumoral H_2_O_2_ generation, such as through enzyme-based strategies using lactate oxidase or glucose oxidase, can reoxygenate hypoxic tumors, activate dendritic cells, and enhance T-cell infiltration, thereby overcoming immunosuppression and sensitizing tumors to radiotherapy and immune checkpoint inhibitors like anti-PD-1 therapy [237–241]. This approach leverages H_2_O_2_ to remodel the TME from immunosuppressive to immunogenic. Conversely, chronic, pathological overproduction of non-radical ROS is a well-established driver of tumorigenesis and cancer progression. In the TME, excessive H_2_O_2_ can promote cancer cell proliferation, survival, and metastasis by persistently activating pro-oncogenic signaling pathways such as NF-κB and HIF-1α, inducing DNA damage that leads to genomic instability, and fostering an immunosuppressive milieu that aids tumor immune evasion [199].

This duality extends to other health domains such as neurodegeneration, where while physiological H_2_O_2_ is crucial for synaptic plasticity and memory formation, its excess contributes to neurodegeneration (Fig. 5b). For instance, an astrocyte-to-neuron H_2_O_2_ signaling cascade was shown essential from a *Drosophila* model for long-term memory formation that was impaired in Alzheimer’s syndrome, underscoring the critical role of physiological level of non-radical ROS in cognition [200]. In another study using *C. elegans* as the modeling biomaterial, mitochondrial H_2_O_2_ triggered neuropeptide release that upregulated organism-wide antioxidant defenses, illustrating circuit-level redox communication [177]. Rat hippocampal slice experiments showed that low μM H_2_O_2_ modulated synaptic plasticity by shifting the long-term potentiation (LTP)/long-term depression (LTD) threshold, while higher concentrations of H_2_O_2_ suppressed transmission, demonstrating concentration-dependent regulation of neural function [247].

Additionally, non-radical ROS have been tightly linked to aging, which is characterized by a critical shift from precise physiological signaling to a state of progressive redox dyshomeostasis. The central mechanism underpinning this transition is the age-related decline in key antioxidant response pathways, most notably the NRF2-Keap1 system. As NRF2 transcriptional activity diminishes with age, the cell’s ability to mount an appropriate defense against baseline non-radical ROS such as H_2_O_2_, is compromised [8]. This decline effectively lowers the damage threshold, meaning that physiological levels of ROS that were once safely buffered and relayed for adaptive signaling are now more likely to cause cumulative oxidative damage. This concept has been directly supported by redox proteomics, which revealed the functional decline of the primary non-radical signal transduction machinery, the PRX-Trx relay; with age, peroxiredoxins, which normally function as high-affinity sensors and transducers of H_2_O_2_, increasingly underwent hyperoxidation and exhibited altered oligomeric states [36, 117]. The transition from beneficial to harmful effects is governed by the efficiency of cellular antioxidant buffers like the NRF2 pathway and the PRX-Trx relay. An age-related decline in these systems, termed ‘non-radical drift’, narrows the protective window for oxidative eustress, making tissues more susceptible to both the pro-cancerous effects of chronic oxidative stress and other age-related pathologies [248, 249]. Therefore, the association of non-radical ROS with disease is fundamentally dose- and context-dependent, positioning them as critical but double-edged players in cellular homeostasis and pathogenesis.

In the realm of tissue regeneration, the therapeutic application hinges on replicating the delicate, low-dose signaling role of endogenous H_2_O_2_. It has been established that a rapidly formed, tissue-scale H_2_O_2_ gradient is an essential early regenerative signal, directing leukocyte chemotaxis to injury sites as shown in zebrafish models [199]. This gradient is crucial for processes like sensory axon regeneration in cutaneous wounds, where epithelial DUOX-derived H_2_O_2_ guides repair, and for promoting keratinocyte migration via IKKα sensing, processes disrupted by antioxidant treatment [201, 250]. The translation of this principle is evident in murine studies, where low-dose topical H_2_O_2_ accelerates wound closure, while high doses are detrimental, defining a strict therapeutic window [202]. This window has been validated in the clinical studies (NCT04771819), as seen with stabilized HOCl improving healing, mirroring the foundational role of endogenous H_2_O_2_ signaling [161].

Lastly, the mechanisms of non-radical ROS have been leveraged therapeutically for treating infection (Fig. 5b). For combating infection, stabilized forms of non-radical oxidants have proven clinically effective. A randomized human trial demonstrated that stabilized HOCl irrigation significantly reduced microbial burden while simultaneously enhancing re-epithelialization in acute wounds, confirming its dual antimicrobial and pro-healing properties at a calibrated dose [161]. Similarly, antimicrobial photodynamic therapy (PDT), which generates ^1^O_2_ at targeted sites, has shown efficacy in eradicating localized infections; randomized clinical trials in periodontitis have confirmed that adjunct PDT improves clinical outcomes like probing depth and reduces key pathogens as compared with standard care alone (NCT05447026) [162, 163].

## Therapeutic strategies targeting ROS

### Antioxidant strategies

#### Radical-focused strategies

Traditional antioxidant strategies were fundamentally rooted in a simplistic, one-size-fits-all ‘scavenging’ strategy (Tables 1 and 2). This approach operated on the core premise that all ROS, particularly radical forms, were uniformly harmful metabolic byproducts responsible for cellular damage and disease. Consequently, the primary therapeutic goal was their global, non-selective neutralization through the administration of high-dose, direct-acting antioxidant compounds. This strategy was exemplified by the widespread use and supplementation of exogenous molecules such as vitamin C (ascorbate) [152, 153], vitamin E (α-tocopherol) [153], N-acetylcysteine (NAC) (NCT1530646) [154] and β-carotene [155], which were intended to donate electrons and quench radicals throughout the body in a stoichiometric manner. The underlying assumption was that boosting systemic antioxidant capacity would linearly correlate with improved health outcomes by reducing the so-called ‘oxidative stress’ burden. This conception has dominated nutritional and therapeutic recommendations for decades, leading to the broad, prophylactic use of antioxidant supplements in the general population without regard for individual redox status or the nuanced physiological roles of ROS as signaling molecules.

The paradigm for radical-focused antioxidant strategies has fundamentally shifted from indiscriminate scavenging to a nuanced, context-dependent approach that acknowledges the essential signaling role of low-dose radical ROS. This strategy is predicated on the recognition that transient, localized bursts of radicals, such as mitochondrial •O_2_^−^ or NADPH oxidase-derived radicals, are not merely toxic byproducts but critical endogenous signaling molecules that drive beneficial adaptations, including enhanced mitochondrial biogenesis, improved glucose metabolism, and elevated cellular stress resistance through pathways like AMPK and NRF2 [87, 106, 154, 178, 229, 251]. For example, mitochondrial ROS (mtROS) directly sulfenylated uncoupling protein 1 (UCP1) in brown adipose tissue, leading to promoted thermogenesis, energy expenditure and consequently loss of weight [86]. Additionally, mitochondrial •O_2_^−^ triggered Ras GTPase (RAS)-mediated signaling and eventually prolonged longevity, directly linking redox modulation to lifespan extension [178].

This hormetic principle is exemplified by exercise, where NOX2-dependent radical pulses like those generated during high-intensity interval training (HIIT) have been shown essential for training adaptations [179]. Also, exercise-induced radicals activated the NRF2/ARE pathway, and the absence of NRF2 eliminated the protection of radicals against cardiac injury [176], underscoring a causative rather than correlative redox mechanism. However, since many adaptive benefits of physical exercises rely on transient radical pulses, high-dose antioxidant supplementation may impede physiological adaptations [106, 156, 157]. For instance, vitamin C (1000 mg/day) combined with vitamin E (235–400 IU/day) has been shown to impair endurance training-induced improvements and inhibit typical health-promoting responses in humans, as demonstrated in double-blind randomized controlled trials (NCT00638560) [156, 157]. While glucose restriction extended the lifespan of *C. elegans*, the effect was abolished by supplementing antioxidants [106].

However, the beneficial impact of radicals on human health is profoundly dose-dependent. That is, while controlled low radical doses can induce adaptive and protective responses beneficial for human health, high levels of oxygen radicals may drive base oxidation, DNA strand breaks, and lipid peroxidation for amplified inflammation and cell death [252]. Thus, precise spatiotemporal control of low concentrations of radicals is required to achieve desirable therapeutic outcome within a safe and beneficial window [253].

Consequently, contemporary antioxidant strategies prioritize redox homeostasis over radical elimination, advocating for personalized intervention rather than universal supplementation. This involves identifying individuals with a demonstrably compromised antioxidant status (e.g., low GSH levels) who may benefit from targeted support while avoiding interference with beneficial radical signaling in healthy individuals [154]; and precise spatiotemporal control of low-concentration radicals within beneficial physiological levels. The therapeutic aim, therefore, is not to suppress all radical activity but to precisely modulate the redox landscape, preventing pathological accumulation that drives damage such as lipid peroxidation in disease while preserving the physiological signaling crucial for metabolic health, stress adaptation, and longevity [106, 252, 253]. This refined strategy represents a move from antioxidant therapy to redox-modulatory medicine, seeking to correct specific imbalances without disrupting essential redox communication networks.

#### Non-radical focused strategies

A non-radical redox strategy leverages diffusible, enzymatically regulated oxidants such as H_2_O_2_ as core signaling molecules that enable cellular adaptation to external stimuli (Fig. 5a, Tables 1 and 2). This process typically involves dose-dependent activation of transcriptional programs. For example, single-cell live imaging has revealed that low levels of H_2_O_2_ initially triggered p53-mediated antioxidant and survival responses, while higher or sustained doses stimulated Forkhead box O1 (FOXO1) signaling, illustrating how signal duration and intensity could shape cells’ adaptive outcomes [116]. This plasticity has suggested a broad therapeutic potential for non-radical oxidants, where delivery protocols can be precisely tuned to induce desired physiological effects. For instance, acute endurance exercise can elevate mitochondrial H_2_O_2_ in skeletal muscles, triggering p53 translocation to the mitochondria. There, p53 forms a complex with mitochondrial transcription factor A (TFAM) and mtDNA to initiate transcriptional programs that drive mitochondrial biogenesis and antioxidant defenses, key adaptations underlying the benefits gained during physical exercise training [203, 254]. On the other hand, lowering carbohydrate availability can enhance reliance on endogenous oxidative signals during exercise, amplifying p53 activation and subsequent mitochondrial gene expression, demonstrating how naturally occurring, transient H_2_O_2_ pulses can be harnessed to potentiate adaptive remodeling [255]. However, prolonged or elevated H_2_O_2_ exposure may shift such a signaling from the p53 to the FOXO1 branch, leading to health concerns such as obesity. For instance, fatty acids-induced oxidative stress in adipose tissue, if without deliberate control, has been reported capable of reshaping energy metabolism and insulin sensitivity by activating FOXO1-dependent pathways, leading to metabolic maladaptation [204, 208, 209].

Additionally, it has been shown that sleep deprivation accelerates aging through disrupting the redox homeostasis dominated by non-radicals. Molecularly, healthy tissues operate on a 24-h redox rhythm, e.g., human red blood cells exhibit peroxiredoxin oxidation cycles that are independent of transcription, indicating that cells anticipate rhythmic ROS/H_2_O_2_ signals throughout the day [256]. Disruption of this rhythm such as through mild sleep restriction could lead to accumulated oxidative stress and impaired redox clearance in freshly isolated human endothelial cells, providing direct evidence that sleep loss could disrupt the vascular radical balance [257]. Among free-living adults with overweight who routinely experienced insufficient sleep, extending sleep duration reduced ad libitum energy intake and produced a negative energy balance, which is consistent with the idea that sleep recovery contributes to the stabilization of daily redox homeostasis (NCT02253368) [160].

### Pro-oxidant strategies

#### Radical-focused strategies

Radical-focused pro-oxidant strategies represent a distinct therapeutic paradigm that deliberately exploits the fundamental chemical properties of ROS, their extremely high reactivity, short diffusion lengths, and capacity for localized, diffusion-limited, and often irreversible chemical reactions, to induce precise and potent cytotoxicity at the nanoscale to submicrometer scale (Fig. 5b, Tables 1 and 2). These therapies function by generating a concentrated burst of radicals in situ, causing catastrophic and clustered damage to critical cellular components like DNA and lipids. A canonical and clinically foundational example is radiotherapy, where ionizing radiation triggers ultrafast water radiolysis to produce •OH within femtosecond to nanosecond timescales. These •OH radicals then react at near diffusion-limited rates to create oxygen-dependent clustered DNA double-strand breaks, which are a primary driver of tumor cell death [258, 259]. A central challenge and a key area of modern refinement is the inherent oxygen dependency of this radical-mediated damage, which limits efficacy in hypoxic tumors. Clinical strategies are actively addressing this limitation. For instance, a randomized phase II study utilized dynamic hypoxia imaging ([^18^F]FMISO PET/CT) to guide and escalate radiation dose specifically to hypoxic subvolumes in head and neck cancer, demonstrating the feasibility and prognostic value of such image-guided, radical-focused dose painting (NCT02352792) [159]. Complementary approaches aim to enhance tumor oxygenation directly, such as using oxygen microbubbles combined with therapeutic ultrasound to improve intratumoral oxygen partial pressure (pO_2_) and thereby boost radical yields during irradiation [191, 192].

Beyond overcoming hypoxia, contemporary radical-focused strategies are innovating to enhance tumor-selective damage through novel mechanisms. FLASH radiotherapy, which delivers radiation at ultra-high dose rates, exemplifies this evolution. Preclinical studies indicate that FLASH irradiation not only spares normal tissues but uniquely drives tumor cell death by significantly increasing lipid peroxidation and inducing ferroptosis. This effect is mechanistically dependent on iron availability, as it is abolished by dietary iron loading, confirming the role of iron-dependent radical chain chemistry in its therapeutic superiority [193]. Another advanced tactic focuses on local radical amplification using high-atomic-number (high-Z) nanoparticle radiosensitizers. A prime example is the intratumoral injection of hafnium oxide nanoparticles (NBTXR3), which, upon irradiation, dramatically amplify the local energy deposit. This leads to severe physical and biochemical damage, including lysosomal membrane permeabilization and subsequent lipid peroxidation, resulting in significantly improved tumor response in preclinical and clinical settings [194].

In summary, radical-focused pro-oxidant therapies are characterized by their reliance on fast, oxygen-modulated, and spatially confined radical reactions to trigger acute cytotoxicity. Modern advancements are strategically focused on conquering the hypoxia barrier and engineering methods to radically amplify damage within the tumor microenvironment, thereby refining this potent cytotoxic approach for greater precision and efficacy.

#### Non-radical focused strategies

Non-radical-focused pro-oxidant strategies represent a sophisticated therapeutic paradigm that deliberately utilizes controlled, diffusible oxidants, such as H_2_O_2_ and its derivatives, to selectively target pathological tissues while minimizing collateral damage, leveraging their predictable chemistry for clinical applications in oncology and infection control (Fig. 5b, Tables 1 and 2).

In cancer therapy, the pharmacological elevation of H_2_O_2_ within tumors is a validated approach, as demonstrated by the pro-drug ascorbate (P-AscH^−^). A clinical study in advanced non-small cell lung cancer (NCT02420314) showed that adding P-AscH^−^ to chemotherapy improved therapeutic response rates, directly supporting H_2_O_2_-mediated targeting as a viable clinical adjunct (NCT02420314) [164]. The mechanistic specificity of this strategy was underscored by the finding that cancer cell resistance was tightly linked to H_2_O_2_ clearance capacity; overexpression of CAT abolished P-AscH^−^ cytotoxicity, and resistant pancreatic cancer cells exhibited enhanced H_2_O_2_ detoxification pathways, positioning antioxidant enzyme profiles (e.g., CAT, PRX-Trx) as critical predictive biomarkers for patient selection [205, 210]. Further precision has been achieved through biomarker-guided stratification, exemplified by a phase II glioblastoma trial (NCT02344355) which correlated iron metabolism markers with patient outcomes under P-AscH^−^-based regimens, enabling a more personalized application [165].

In the realm of infection control and wound care, non-radical oxidants are deployed for their broad, controllable antimicrobial action. For example, vaporized H_2_O_2_ systems were established for room and instrument decontamination, while stabilized HOCl demonstrated dual pro-healing and antibacterial effects in randomized trials for chronic wound management (NCT04771819) [161, 260]. The development of continuous systems like dry H_2_O_2_ for occupied spaces and ozonated water for surface disinfection highlighted the adaptability of these non-radical agents for clinical and environmental sanitation [211–213]. The core strength of these strategies lies in their controllable delivery, minimal residue, and repeatable dosing (NCT02905578) [166, 260]. However, clinical translation faces constraints such as limited tissue penetration depth for light-activated therapies, rapid enzymatic degradation of H_2_O_2_/HOCl, and variable tissue perfusion. Ongoing innovation is therefore directed at overcoming these barriers through advanced delivery formats (e.g., daylight-PDT, dry-fog systems) and refined, biomarker-guided patient stratification to ensure that these potent non-radical pro-oxidant tools achieve maximal therapeutic selectivity and efficacy (NCT03805737) [165, 167, 210, 261].

### Cold atmospheric plasma as a emerging hybrid redox modulation tool

**Fig. 6:**
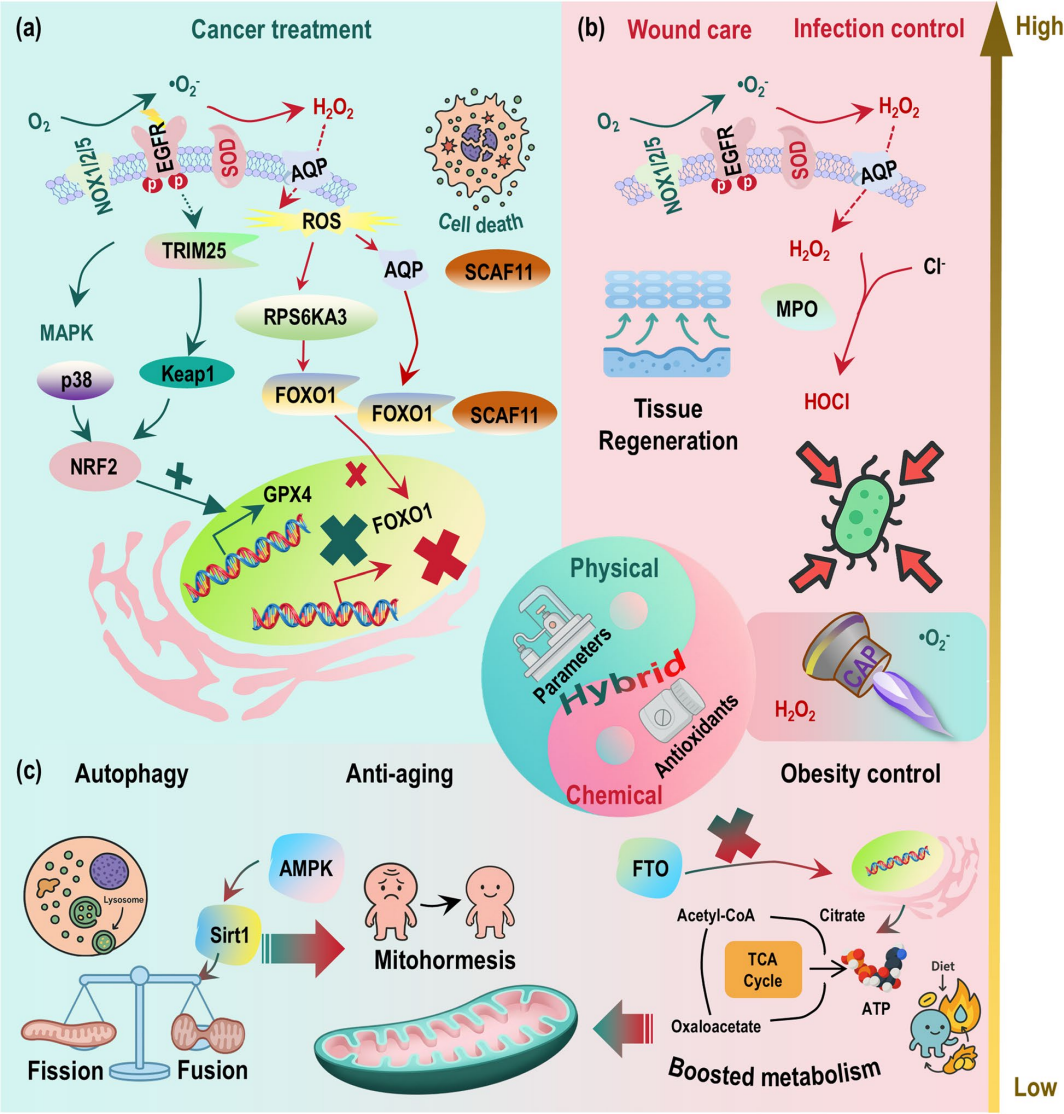
Cold atmospheric plasma as a hybrid of radical and non-radical species for health and disease management. Cold atmospheric plasma (CAP) represents a unique redox modality that simultaneously generates short-lived radical species and longer-lived non-radical oxidants, thereby integrating localized cytotoxic reactions with diffusible, programmable redox signaling. This hybrid chemical composition enables dose- and context-dependent applications spanning disease treatment and health promotion. **a** Cancer treatment. At high operational intensities, CAP generates abundant radical ROS (e.g., •O_2_^−^ and •OH) at the cell surface, inducing localized, nanoscale cytotoxic signaling. Radical species interact with membrane-associated proteins such as EGFR [195] to initiate ferroptotic pathways, whereas non-radical oxidants, primarily H_2_O_2_, enter cells through aquaporins (AQPs) and trigger intracellular apoptotic signaling cascades [266]. Through this dual mechanism, CAP selectively induces cancer cell death while sparing surrounding healthy tissue. **b** Wound care and infection control. At therapeutic doses, CAP-derived non-radical ROS dominate biological outcomes. Diffusible H₂O₂ promotes tissue regeneration by supporting redox-controlled repair processes, while in immune contexts, myeloperoxidase (MPO) converts H_2_O_2_ into hypochlorous acid (HOCl), enabling efficient and broad-spectrum antimicrobial activity. These effects collectively facilitate wound healing and infection control without excessive tissue damage. **c** Health promotion and metabolic regulation. At low intensities, CAP delivers a balanced mixture of radical and non-radical ROS that induces adaptive redox signaling rather than cytotoxicity. This low-dose redox input activates AMPK-dependent autophagy, promotes mitochondrial remodeling and mitohormesis, and inhibits nuclear entry of FTO, thereby enhancing metabolic efficiency. These mechanisms underpin the potential applications of CAP in anti-aging and obesity control. Overall, CAP functions as a tunable hybrid redox platform in which radical ROS dominate near-field, high-dose cytotoxic effects, while non-radical ROS mediate long-range, low-dose signaling for regeneration and metabolic adaptation. The relative contribution of each ROS class is precisely controlled by physical operating parameters and chemical modulators such as antioxidants. Dark blue and dark red represent radical and non-radical ROS at relatively high concentrations, respectively. Light gradient color (blue mixed with red) represents both radical and non-radical ROS at relatively low concentrations. Additional abbreviations: AMPK, AMP-activated protein kinase; AQPs, aquaporins; CAP, cold atmospheric plasma; EGFR, epidermal growth factor receptor; FTO, fat mass and obesity-associated protein; ROS, reactive oxygen species

Cold atmospheric plasma (CAP), a fourth state of matter, represents a uniquely versatile therapeutic platform that directly addresses the fundamental dichotomy in redox biology by inherently coupling the generation of short-lived, highly reactive radical species (e.g., •OH, O, •NO) with longer-lived, diffusible non-radical oxidants (e.g., H_2_O_2_, nitrite/nitrate, and ONOO^−^-derived species) in a single, tunable delivery system [5, 195, 196, 259, 262–265]. This co-delivery mechanism enables a unique two-phase action: an immediate, potent ‘radical hit’ confined to the near-field site of application, causing localized and often irreversible biomolecular damage, followed by a sustained, programmable ‘non-radical afterglow’ that diffuses to modulate signaling pathways and inflammatory responses over a broader spatial and temporal range. By simultaneously accessing both ends of the ROS reactivity spectrum, the localized, acute trigger of radicals and the sustained, regulatable signal of non-radicals, CAP achieves a level of multimodal redox intervention unmatched by single-agent therapies, thereby supporting its broad and adaptable application spectrum from promoting wound healing and tissue regeneration to targeting tumors and sterilizing infections (Fig. 6).

#### The radical feature of CAP for disease management

CAP can engage radical chain chemistry at the receptor-proximal sites. For instance, CAP triggered apoptosis and ferroptosis of triple negative breast cancer cells by generating radical ROS and potentiating their interactions with cell surface receptors. Such interactions led to the post-translational modification of receptors such as EGFR(Y992/Y1173) phosphorylation during apoptosis [196] and EGFR(Y1068) phosphorylation during ferroptosis [195] (Fig. 6a). Direct CAP exposure generated membrane •OH and thus triggered HOCl-dependent signaling, and applying mannitol or N,N’-dimethylthiourea (DMTU), both are scavengers of •OH, abrogated the apoptosis of gastric cancer cells [214].

Delivery systems further refine the distribution of radical species for improved targeting and therapeutic effects in vivo*.* While CAP-activated medium takes actions via mostly diffusible non-radicals, directly exposing lesions with CAP using, e.g., *invivo*Pen, exposed the cancer tissues with more near-field radicals. Though both produced stable, reproducible cancer cell killing performance with inevitable adverse effects [197], radicals have been considered to be more feasible for treating complex syndromes such as cancers [195, 196], whereas non-radical components take the leading roles in applications such as disinfection [267, 268].

#### The non-radical feature of CAP for disease management

In addition to the predominant roles of radicals in oncology, non-radicals such as H_2_O_2_ can enter cells and mediate intracellular redox signaling for cell killing. For example, CAP suppressed the stemness of triple-negative breast cancer cells via modulating AQP3-mediated AQP3-5 K and FOXO1 K48-ubiquitination, where H_2_O_2_ entry through AQP3 for signal relay played a significant role [266] (Fig. 6a). Consistent with this non-radical paradigm, plasma-oxidized saline produced an H_2_O_2_-dominant, long-lived afterglow in peritoneal carcinomatosis models; and the same cytotoxic effect was observed when exposing cancer cells to solutions enriched with H_2_O_2_ and NO_3_^−^, supporting the inevitable role of diffusible long-lived species in resolving cancers [215]. In orthotopic glioblastoma models, non-invasive CAP sensitized tumors to temozolomide and improved disease control compared to temozolomide alone [216]. CAP-activated saline suppressed melanoma xenograft growth in vivo and altered tumor metabolite profiles, indicating the efficacy of CAP as a stand-alone antitumor therapy [217]. Additionally, CAP-activated saline combined with low-dose cisplatin reduced primary tumor growth and metastasis with minimal toxicity in an orthotopic ovarian cancer model, suggesting their synergistic activity in rewiring drug resistance [218]. Repeated topical CAP application also impeded the progression of UVB-induced cutaneous squamous cell carcinoma in mice without damaging surrounding healthy tissue [206]. Mechanistically, CAP-activated medium induced ferroptotic cell death accompanied by lipid ROS accumulation in lung cancer cells, providing a molecular basis for CAP’s antitumor effects [198]. Clinically, a first-in-human phase I study confirmed that intra-operative CAP was feasible and well-tolerated in patients with advanced solid tumors during surgical resection, supporting its clinical use for margin control (NCT04267575) [169].

Besides, CAP has been applied for wound care and infection control, largely due to its non-radical components. In chronic venous leg ulcers, adjunctive direct CAP treatment administered once or twice weekly significantly enhanced healing rates compared to standard care alone in a randomized controlled trial (NCT04922463) [170]. Similarly, a randomized placebo-controlled trial in diabetic foot ulcers demonstrated that CAP accelerated wound area reduction and shortened time to meaningful closure, irrespective of baseline infection status (NCT04205942) [171]. CAP also resulted in an immediate reduction in bacterial load in a randomized clinical trial in treating diabetic foot ulcers, correlating with improved wound closure outcomes (IRCT20080904001199N2) [172]. For pediatric verruca vulgaris, in a murine model of oral mucositis with candidiasis following chemotherapy/radiation, CAP jet treatment reduced fungal burden and prevented systemic dissemination, highlighting its anti-infection potential in treating mucocutaneous inflammation [219]. A randomized trial reported that CAP achieved complete clearance rates equivalent to cryotherapy, alongside significantly reduced post-procedural pain (NCT05070754) [173]. Furthermore, a prospective study indicated that CAP exhibited antifungal efficacy and clinical improvement comparable to oral itraconazole in treating *Malassezia folliculitis* (NCT04886323) [174].

#### The hybrid feature empowers CAP with duality in disease management

The hybrid radical/non-radical spectrum of CAP makes it possible to treat diversified types of syndromes such as cancers and autoimmune diseases [263]. Taking cancer as an example, CAP can target all hallmarks of transformed cells via post-translational editing [269, 270] or signal relay intervention [271].

Beyond the life and death control over malignant cells where the coordination between radicals and non-radicals has been previously emphasized [266], it is of the particular significance to underscore the unique duality of CAP in reshaping the tumor-immune interface and abnormal metabolism. For instance, while longer-lived non-radical components of CAP primarily induced macrophage M1 polarization, radical bursts provided the initial danger signals [272]. This has made it possible to integrate CAP into the immunotherapeutic modality for reinforced efficacy and minimized adverse effects. As one example, applying CAP eliminated immunosuppressive pancreatic stellate cells and triggered immunogenic death (ICD) of pancreatic cancer cells [220], and coupling CAP with anti-PD-1 augmented intratumoral immune activation and, consequently, improved therapeutic efficacy [221]. From the perspective of metabolism, CAP potentiated high-rate apoptosis of chronic myeloid leukemia cells, primary CD34^+^ hematopoietic stem and progenitor cells, while sparing their normal counterparts by disrupting the skewed metabolism of tumor cells as a result of ultra redox sensitivity [222].

Taken together, CAP functions as a programmable hybrid modality (Fig. 6). While radicals enable rapid, localized, irreversible killing via, e.g., EGFR-GPX4-mediated ferroptosis, non-radicals provide diffusible, enzyme-modulated control over cell signaling. This architecture supports CAP as a promising anti-cancer selective monotherapy, as well as the rational of combining it with immune or metabolic agents, with delivery-informed expansion of the therapeutic window [180, 195, 196, 263, 273].

Last but not the least worthy to be addressed is the radical/non-radical duality of CAP represented by its host–pathogen interactions. While CAP prevented ACE2-mediated virus entry by internalizing and downregulating ACE2, a receptor in response to radical ROS potentiated signaling. In contrast, once non-radical components of CAP entered cells, they can enhance viral replication via inducing EGFR (Tyr1068)-mediated mitophagy, highlighting how differential types of ROS determine whether CAP supports defense or facilitates infection [265, 274].

#### Dose calibration for controlled hybrid feature of CAP in disease management

It is noteworthy to emphasize the selectivity of CAP in killing transformed cells without harming their healthy peers on appropriate dosage. For instance, CAP selectively inhibited prostate cancer cells through triggering G_0_/G_1_ arrest and apoptosis with minimal impact on non-malignant cells, and exhibited specificity against triple negative breast cancer cells both in vitro and in vivo [223, 224]. At a broader scope, CAP could promote oxidative stress and induce death in transformed cells at higher doses; and upregulate antioxidant machinery, thereby supporting normal cellular signaling at lower doses [263, 275]. Such a multifarious nature of CAP has enabled its diversified applications in disease management, yet should be under appropriate control before it can be safely applied to the clinics for desirable outcomes [276–279]. Luckily, its calibrated control can be achieved from both physical and chemical perspective, in principle. From the physical point of view, the dosing of CAP can be controlled over its discharge mode, power, feed gas, and surrounding medium to align with the specific clinical setting [263, 264, 273, 280, 281]. From the chemical aspect, the biphasic property of CAP can be well harnessed by antioxidants or their counteractive inhibitors for buffered effects and constrained therapeutic window (Fig. 6). For instance, spermidine counteracted CAP’s anti-tumor action by inducing transient G₀/G₁ arrest in triple negative breast cancer cells, underscoring the need for biomarker-guided scheduling to avoid antioxidant interference in redox-based therapeutics [180]. Auranofin (a TrxR inhibitor), on the other hand, synergized with CAP in triggering distinct death programmes of glioblastoma cells [225].

To enable species-matched dosing, multi-omics integration can be used to inform the selection of CAP ejection parameters and possible combination strategies. For this purpose, CAPmed-BC, the first and sole web service integrating multiple high throughput omic data including whole transcriptome, proteome, phosphoproteome, acetylome, and lactylome of breast cancer cells in response to CAP treatment, can be used. It can assist decision making in when to favor non-radical signaling (e.g., through AQP3-mediated H_2_O_2_ cell entry and signal relay) versus radical execution (e.g., EGFR-potentiated cell ferroptosis) regarding the parameter selection when treating a specific patient given its molecular profiles [264].

#### CAP for health management

Beyond its applications in disease treatment, CAP can be utilized in health management as a dose-controlled, spatially confined, low-intensity stimulus that modulates redox homeostasis in skin and metabolic tissues without causing structural damages. This capability supports CAP for anti-oxidation, anti-aging, and body management including obesity control (Fig. 6c).

In a photoaging model, low-intensity CAP significantly reduced visible wrinkles and up-regulated dermal extracellular matrix (ECM) proteins, and these effects persisted for several weeks, indicating adaptive tissue remodeling rather than destructive damage [181]. On intact healthy skin, brief exposures to dielectric barrier discharge CAP enhanced local microcirculation and tissue oxygenation with precise spatial confinement, and the improvements persisted for approximately one-hour post-treatment, offering measurable indicators of immediate tone and recovery enhancement [158, 168]. For daily skin care, CAP suppressed lipogenesis and proliferation in human sebocytes in vitro and reduced facial sebum production in vivo without causing thermal injury, supporting the use of CAP in oil-control, acne-prone, and anti-shine-aging strategies [175]. In post-exercise recovery, CAP accelerated skeletal muscle repair and ECM remodeling in vivo, suggesting a potential role for CAP within exercise-recovery regimens [182]. CAP inhibited adipogenesis and triglyceride accumulation in 3T3-L1 preadipocytes, downregulated a spectrum of lipogenic markers and, importantly, reduced adipose tissue mass in high-fat-diet-fed mice without affecting food intake, suggesting that CAP can be safely applied for body weight management by affecting the metabolism [183]. Mechanically, this has been molecularly linked to *FTO*, a gene encoding fat mass and obesity-associated protein, where CAP has been proposed for regulating fat metabolism and energy homeostasis as an innovative type of FTO inhibitor [282]. In addition, human adipose-derived stem cells maintained viability following exposure to low-dose CAP-activated solution without losing phenotypic properties, defining a translational window of safety and efficacy for combined CAP-based and biologic or material-assisted approaches in subcutaneous tissues that can be used for contour maintenance [283, 284].

## Biomarker-guided stratification for species-oriented therapy

Species-oriented therapy should stratify tumors based on three measurable axes, i.e., oxidant production, antioxidant buffering, and transmembrane transport, as these have collectively determined whether radical chemistry induces damage or non-radical flux supports signaling. Markers thus can be briefly categorized into buffering markers, transportation markers, and bioactivation markers indicative of, e.g., oxygenation and iron status.

Buffering markers are clinically actionable across both radical- and non-radical-oriented regimens. For example, elevated TXNRD1 was correlated with anti-PD1 resistance in head and neck cancer [285]. TrxR inhibition with auranofin restored therapeutic sensitivity of cancer cells to radical-potentiated ferroptosis [285]. In the model of non-small cell lung cancer, auranofin synergized with CHK1 inhibition and resulted in enhanced radical-mediated killing by disrupting ribonucleotide reductase regulation and connecting thiol buffering to DNA damage response [285]. Buffering also modulated responses to non-radical oxidants. The Keap1-NRF2 pathway serves as a key indicator of buffering capacity against non-radical H_2_O_2_ and often predicts resistance to oxidative therapies [286]. Noninvasive redox imaging has advanced significantly. For example, [^18^F] FSPG PET was used to visualize NRF2-mediated system xₙc^−^ activity and anticipate therapeutic resistance, providing a selection tool for H_2_O_2_-based therapeutic strategies including possibly CAP as well in non-small cell lung cancer management [287]. Early clinical data revealed heterogeneous [^18^F] FSPG uptake across tumors and patients, supporting its use in both patient selection and intra-tumor targeting [287]. In addition, exogenous CAT rescued lung cancer cells from pharmacologic ascorbate cytotoxicity by scavenging H_2_O_2_, identifying CAT/PRX-Trx capacity as a resistance biomarker [288].

Transport in-depth defines the spatial reach of non-radical species and the ‘afterglow’ component of hybrid modalities like CAP. Peroxiporins such as AQP3/8 regulated H_2_O_2_ membrane permeability and modulated EGFR-PI3K-AKT signaling, suggesting that AQP expression may help select patients for H_2_O_2_-focused therapies or CAP regimens with pronounced non-radical afterglow [266].

Radical-focused strategies require oxygen supply, as hypoxia can restrict radical propagation and radiosensitization. Additionally, cells’ susceptibility to ferroptosis offers another stratification layer. For example, the levels of SLC7A11/GPX4 were positively correlated with the progression of colorectal cancer, whereas inhibiting this axis via targeting FTO induced ferroptosis [289]. Bioactivation markers can further refine non-radical strategies. For example, NQO1-high tumors were sensitive to β-lapachone, which exhibited chemopreventive efficacy against lung premalignancy in vivo, indicating a potential role for H_2_O_2_-releasing prodrugs in prevention or adjuvant therapy [290]. For high-dose ascorbate, iron status was used to predict the therapeutic response, where T2*MRI of tumor iron metabolism identified responders in a phase II glioblastoma trial (NCT02344355) [165]. Additional clinical studies proposed iron-focused panels to personalize dosing and combinations, aligning non-radical tactics with measurable iron availability [291].

Collectively, these biomarkers may bridge chemistry and clinic by aligning radical or non-radical mechanisms with a tumor’s detoxification capacity, transport properties, bioactivation markers indicative of, e.g., oxygenation and iron status, enabling more precise and efficient patient stratification [289, 292].

## Conclusions

ROS biology can be best understood through two interconnected chemical paradigms. The first paradigm distinguishes spatial and kinetic behaviors. While short-range, rapid radical reactions establish local thresholds and induce acute damage, longer-range, buffered non-radical fluxes propagate and integrate signals via PRX-Trx relays and peroxiporin-mediated transport to support physiological plasticity. This explains why radicals act proximally to their origin, whereas non-radicals traverse membranes to form gradients enabling specific signaling. The second paradigm is the dose-dependent duality of both radical and non-radical ROS. While both types of ROS mediate adaptive oxidative eustress at low physiological levels, they drive pathogenic oxidative distress at high concentrations, contributing to the pathogenesis of various health concerns like aging and diseases like cancer.

From the translational point of view, species-matched redox strategies represent a paradigm shift, moving from nonspecific antioxidant supplementation toward the precise targeting of distinct ROS to harness their signaling or cytotoxic functions. For health maintenance, low-dose, pulsed radical species (e.g., from exercise) act as essential signaling mediators that potentiate adaptive responses in metabolism and mitochondrial homeostasis, benefits contingent upon strict dose control. Non-radical oxidants serve as versatile, dose-dependent regulators of cellular plasticity, whose functions are deeply tied to systemic rhythms like circadian redox oscillations; disruption of these rhythms (e.g., via sleep loss) can accelerate aging and metabolic dysregulation, highlighting the need for temporally precise interventions. For disease treatment, species-matched therapeutics exploit distinct mechanisms. While radiotherapies primarily leverage radical-mediated killing (dependent on oxygen and iron), agents like pharmacologic ascorbate largely utilize diffusible non-radicals for programmable selectivity. On the other hand, precise dosage control is of critical importance, as it is fundamentally governed by the endogenous antioxidant capacity of the target cells. The robustness of these cellular buffering systems such as the GPX4-SLC7A11 axis and the Keap1-NRF2 pathway determines the sensitivity to exogenous redox perturbations and dictates the ultimate biological outcome. This principle is exemplified in pathology. That is, tumors exhibiting high expression of GPX4-SLC7A11 were associated with a poor prognosis [289], as this elevated their threshold for radical-mediated ferroptosis. Similarly, chronic activation of NRF2, while protective under stress, led to therapy resistance by permanently raising the cellular detoxification ceiling, creating a fortified redox state [10, 286]. These observations argue decisively against blanket antioxidant suppression, which could inadvertently bolster such resistant phenotypes. Instead, they favor strategic interventions that either preserve beneficial, transient ROS signaling windows or, conversely, employ dose-time–space targeted delivery of pro-oxidant fluxes to overwhelm specific antioxidant defenses in pathological cells. Thus, the maintenance of health and the control of disease both hinge on a nuanced understanding and precise manipulation of the redox homeostasis orchestrated by the interplay of radical and non-radical species.

This highlights the unique advantage of integrating both radical and non-radical species into a coordinated strategy. In this context, CAP has emerged as a highly promising modality, as it synergistically delivers an initial radical-mediated ‘hit’ followed by a sustained non-radical ‘afterglow’. This dual-phase action has demonstrated efficacy across diverse clinical conditions. We particularly emphasize the distinct and non-substitutable roles of radical species in treating complex diseases like cancer, where their functions extend beyond microbial killing and cannot be replicated by non-radical oxidants. This may foster an innovative field, plasma complex medicine, where the controlled, sequential delivery of distinct ROS enables precise targeting of pathological mechanisms and tissue regeneration. Advancing this field will require a biomarker-guided approach, incorporating metrics such as NRF2 activity ([^18^F]FSPG PET), peroxiporin expression, GPX4-SLC7A11 status, iron levels (T2*MRI), and tissue oxygenation (OE-MRI/[^18^F]FMISO-PET) to tailor context-specific dosing and therapeutic combinations, thereby accelerating clinical translation.

In summary, this paper has established a dual-paradigm framework for ROS in biology and medicine, distinguishing between the spatial-kinetic behaviors and the dose-dependent duality of radical versus non-radical species. This framework logically culminates in the concept of species-matched redox interventions, which aim to precisely harness or inhibit specific ROS for therapeutic benefit. The innovative potential of integrated approaches, exemplified by CAP and guided by advanced biomarkers, naturally leads to a critical examination of the current clinical evidence and future trajectories for these strategies.

## Data Availability

Not available.
